# The Power of Yeast in Modelling Human Nuclear Mutations Associated with Mitochondrial Diseases

**DOI:** 10.3390/genes12020300

**Published:** 2021-02-20

**Authors:** Camilla Ceccatelli Berti, Giulia di Punzio, Cristina Dallabona, Enrico Baruffini, Paola Goffrini, Tiziana Lodi, Claudia Donnini

**Affiliations:** Department of Chemistry, Life Sciences and Environmental Sustainability, University of Parma, Parco Area delle Scienze 11/A, 43124 Parma, Italy; camilla.ceccatelliberti@unipr.it (C.C.B.); giulia.dipunzio@unipr.it (G.d.P.); cristina.dallabona@unipr.it (C.D.); enrico.baruffini@unipr.it (E.B.); paola.goffrini@unipr.it (P.G.); tiziana.lodi@unipr.it (T.L.)

**Keywords:** yeast model, mitochondria, diseases

## Abstract

The increasing application of next generation sequencing approaches to the analysis of human exome and whole genome data has enabled the identification of novel variants and new genes involved in mitochondrial diseases. The ability of surviving in the absence of oxidative phosphorylation (OXPHOS) and mitochondrial genome makes the yeast *Saccharomyces cerevisiae* an excellent model system for investigating the role of these new variants in mitochondrial-related conditions and dissecting the molecular mechanisms associated with these diseases. The aim of this review was to highlight the main advantages offered by this model for the study of mitochondrial diseases, from the validation and characterisation of novel mutations to the dissection of the role played by genes in mitochondrial functionality and the discovery of potential therapeutic molecules. The review also provides a summary of the main contributions to the understanding of mitochondrial diseases emerged from the study of this simple eukaryotic organism.

## 1. Introduction

Mitochondrial diseases (MDs) are inherited disorders that, through various mechanisms, lead to mitochondrial dysfunction. The genetic cause of MDs includes mutations in either mitochondrial DNA (mtDNA) or nuclear DNA genes. Due to the dual genetic control of mitochondrial function (nuclear and mitochondrial), MDs display different inheritance pattern: sporadic, maternal, autosomal dominant, autosomal recessive or X-linked [1].

Mitochondria provide energy to cells by oxidative phosphorylation (OXPHOS) carried out by a series of multi-heteromeric complexes embedded in the mitochondrial inner membrane. These complexes constitute the mitochondrial respiratory chain (MRC) that, through sequential reactions of reduction and oxidation, performs “cellular respiration” [2]. The OXPHOS process is responsible for the supply of energy to cells; any defect or alteration in the process leads to pathological consequences, mainly in tissues and organs that have a high energetic request such as the brain, skeletal muscles, and heart. The consequences of an impairment of the OXPHOS system are a decrease in ATP production and an increase in reactive oxygen species (ROS). Mitochondria are mainly involved with the production of ATP by OXPHOS and play a role in other bioenergetic pathways such as the tricarboxylic acid cycle (TCA)—[3] and fatty acids β-oxidation [4]. In addition to these primary functions, mitochondria are also involved in biosynthetic pathways, including but not limited to amino acids and nucleotides [5], iron sulphur cluster [6], and in cell signalling with a determinant role in apoptosis [7], and calcium homeostasis [8].

Given the complexity of mitochondrial genetics and biochemistry, mitochondrial inherited diseases may present extremely heterogeneous clinical manifestations, ranging from lesions in single tissues, such as the optic nerve in Leber’s hereditary optic neuropathy, to more diffuse lesions including myopathies, encephalomyopathies, cardiopathies and hepatopathies, all the way to complex multisystem syndromes characterised by a vast range of symptoms, severity, age of onset and outcome [9,10,11,12]. Genetic defects of OXPHOS, the group of mitochondrial disorders predominantly identified to date, have a prevalence at approximately 1:5000 [13,14,15] making these the most common pathologies with a genetic basis. However, more recently, the term “mitochondrial disease” has also been extended to another series of pathologies, known as secondary mitochondrial dysfunction (SMD), caused by mutations in genes that are not involved in the production or functionality of respiratory complexes [16]. For example, defects in the mitochondrial fission/fusion processes are implicated in the onset of age-related human disease, such as Alzheimer’s and Parkinson [17] or cardiovascular disease [18], thus underlining the pivotal role of the mitochondrion in many cellular functions, beyond energy production. 

To date, thanks also to the increasingly widespread application of the next generation sequencing (NGS) to whole exome sequencing (WES) and whole genome sequencing (WGS), pathogenic variants in approximately 300 disease genes have been described [1,19] most associated with dysfunction of mitochondrial energetics [20]. However, in a large fraction of patients with MDs, the genetic basis is still unknown. This is not surprising since there are approximately 1200 human genes encoding mitochondrial localised proteins [21,22]. 

On the other hand, the identification of numerous nuclear variants of unknown significance requires functional validation to confirm pathogenicity and an in-depth analysis to assess the mechanisms through which they are associated with mitochondrial disease [1].

Model systems (yeast, *Caenorhabditis elegans*, *Drosophila,* zebrafish, mouse) have proved their usefulness to validate the pathogenicity of variants, to assess the disease progression and the mechanisms associated with mitochondrial dysfunction. They therefore represent a powerful tool to study new disease genes, in particular when the gene function is unknown, when there is only a single patient, patient samples cannot be obtained or when cell lines, derived from patient fibroblasts, are aphenotypic (see for recent review [1,20,23]). The yeast *Saccharomyces cerevisiae* is the organism that more than any other has contributed to our understanding of mitochondria functionality. In fact, it was in this organism that the cytoplasmic factor *rho* (*ρ*), later identified with the mtDNA, was initially detected, and its role in the generation of respiratory enzymes was proven [24,25]. *Saccharomyces cerevisiae* is a facultative anaerobe yeast, able to grow on fermentable and non-fermentable (i.e., oxidative) carbon sources. This eukaryote has the peculiarity to survive on fermentable carbon sources in the absence of mtDNA, which makes it a major player in our understanding of the mitochondrial biogenesis. Mutations affecting mitochondrial functions are, in fact, easily identifiable in media containing an oxidative carbon source such as glycerol, ethanol, or lactate. More generally, the success of this experimental single cell organism is linked to its efficient homologous recombination properties, which allowed the creation of genetic knockouts collections [26], to the easiness of its manipulation [27,28] and to the high degree of similarity in cellular activities, including those related to mitochondria, with higher, and more complex, eukaryotes [29,30]. Remarkably, more than 50% among the 1000 protein species estimated in yeast mitochondria [31,32] have a human homolog [21], and 70% of the nuclear genes involved in human mitochondrial diseases are conserved in yeast [20]. Thanks to these features, *S. cerevisiae* has significantly contributed to the identification of the molecular basis of numerous mitochondrial diseases, as reviewed elsewhere [20,33,34,35,36]. The aim of this review was to summarise the advantages that the yeast model offers in the validation of mutations, in the determination of their heritability, in deepening the role of genes and of specific mutations in mitochondrial functionality, in the discovery of potential therapeutic molecules and to give a few examples of their application. In particular, this review intends to highlight the main advantages offered for the diagnostics of mitochondrial disorders caused by mutations in nuclear genes, whose list increases day by day due to the advent and application of NGS technologies. 

## 2. Modelling Putative Disease Variants 

**Figure 1:**
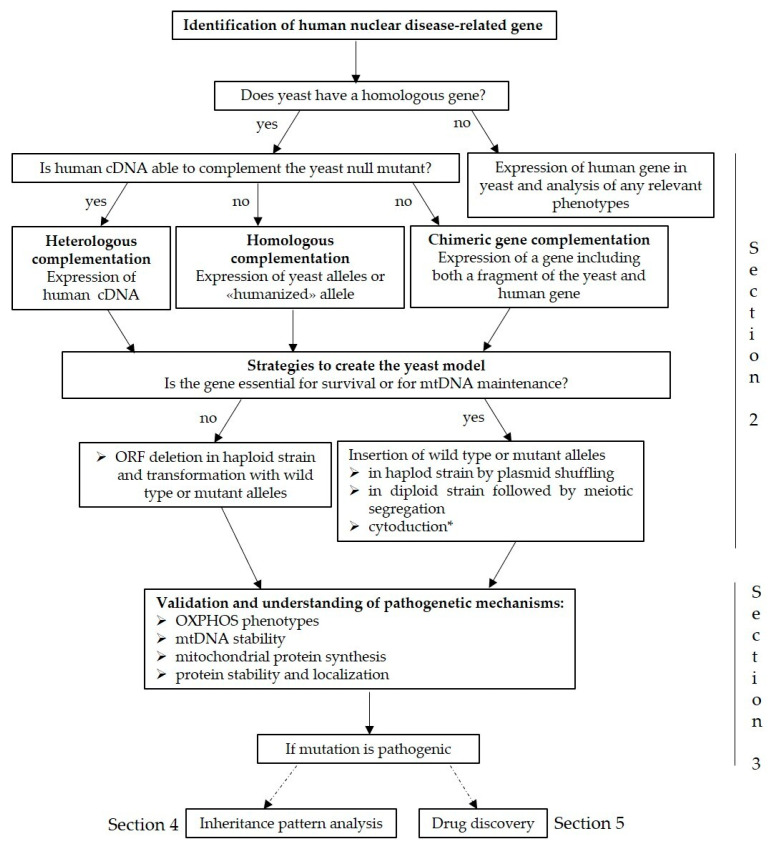
Workflow for the creation of the yeast models of mitochondrial diseases. * cytoduction is a strategy only used in case the gene is essential for mitochondrial DNA (mtDNA) maintenance. cDNA, complementary DNA; ORF, open reading frame; OXPHOS, oxidative phosphorylation.

Considering the huge number of novel genetic variants identified by NGS, a major challenge is to determine if these are the cause of the pathology of interest. Bioinformatic tools can certainly help but they are not sufficient to confirm the causality that must be biologically proven through functional analyses. The experimental workflow changes depending on whether the variants identified are novel and appear in a gene that has previously been associated with disease or in a gene not previously linked to disease and in this case, if the function of the gene is known or not. The workflow for the creation of the yeast models of mitochondrial diseases is represented in Figure 1.

For the creation of the model, the first question to ask is whether yeast has a gene orthologous to the human one. When a human disease-related gene is not present in the yeast genome, the human gene can be expressed in yeast under the control of a regulatable promoter to modulate the levels of the heterologous protein. The analysis of any relevant phenotypes associated to the expressed gene is then performed. Examples of “humanised” yeast models comprise neurodegenerative disorders in which the pathogenesis is associated to protein misfolding with the consequent formation of aggregates or oligomers. Disorders s such as Parkinson’s, Huntington’s and polyglutamine (poly(Q)) diseases have been modelled in yeast and have been extensively documented in the review by Khurana and Lindquist [37].

When a homolog of the gene involved in the disease is present in the yeast genome, heterologous, homologous or chimeric gene complementation approaches can be used depending on the ability/inability of the human cDNA to complement the yeast null mutant strain. As reported in Table 1, to validate the pathogenicity of novel genetic variants, several yeast models of diseases have been constructed using the different approaches.

In order to use the heterologous complementation approach, the human cDNA is inserted in a specific yeast expression vector, under the control of an appropriate promoter, and containing a yeast selectable marker. 

An example of a heterologous complementation approach refers to the GRACILE syndrome-related gene *BCS1L*, which encodes a mitochondrial chaperone required for the correct assembly of complex III being necessary for the incorporation of the Rieske FeS protein Rip1 [46,47,48,50,202]. Another example of validation where human cDNA was directly used is that of *COASY* [165,166], a gene encoding for the mitochondrial bifunctional enzyme, coenzyme A synthase [203], whose mutations are associated with the development of a form of neurodegeneration with brain iron accumulation (NBIA), namely CoPAN (COASY protein-associated neurodegeneration) characterised by iron accumulation in the brain and the impairment of mitochondrial energy generation [165,204]. 

It must be underlined that using the human cDNA to evaluate the consequence of a mutation has the advantage that a direct demonstration of the role of the amino acid substitution is obtained. However, this approach could not be suitable if the complementation is fair, preventing further phenotypic analysis, or if the expression levels are not optimised. 

When human cDNA is unable or unsatisfactorily to complement the yeast null mutant, the homologous complementation approach is performed. This approach is based on the fact that if an amino acid is conserved or semi-conserved, it should perform the same role in yeast and human protein, so that a detrimental effect in the yeast protein should replicate what happens in the human protein. At first, the conservation of the amino acid during the evolution from yeast to human is evaluated by the alignment of proteins. If the residue is conserved, it could be directly mutagenized, thus producing the “pathological” allele. When the mutation affects a non-conserved residue but the surrounding stretch is conserved, a general role of this region is suggested; in this case, it is possible to replace the yeast amino acid with the corresponding wild-type residue present in human protein to serve as the “humanised” control. The yeast mutant allele and the humanised control are then expressed in a yeast strain deleted of the gene under analysis to evaluate the ability to complement the mutated phenotype. In this approach, the gene copy present in the yeast genome can be directly mutagenized, for example, through the “delitto perfetto” technique [205]. Alternatively, the wild-type and mutant genes can be cloned in a plasmid and inserted in the null mutant. An example of homologous complementation approach regards the disease-related gene *ISCU* which encodes a scaffold protein needed for the assembly of iron–sulfur (Fe–S) clusters and whose recessive mutations lead to myopathy or skeletal and cardiac myopathy [206,207,208] in human. The modelling of a heterozygous missense mutation in the corresponding yeast gene *ISU1* allowed to confirm both pathogenicity and dominance of the new variant [162] (see also paragraph 4). The same approach also allowed to validate the pathogenic role of a mutation in *COX6B* gene, encoding a subunit of complex IV, associated to severe infantile encephalomyopathy [43]. 

If the human cDNA does not complement the yeast deletion, and the amino acid under investigation is present in a region which is not conserved, a third strategy can be attempted, based on the construction of a chimeric gene which includes a fragment of the yeast gene and a fragment of the human cDNA. One of the causes of the lack of complementation of genes encoding for mitochondrial proteins is that the human mitochondrial targeting sequence (MTS) necessary for the import into the mitochondria is not recognised by the yeast import machinery, since the MTS sequences are partially different between mammals and yeast [209]. In this case, the chimera can be constructed by changing the region encoding the MTS of the human gene with its yeast counterpart, or with a generic yeast MTS. Such an approach has been used, for example, for constructing a model for studying mutations in *POLG*, encoding the mitochondrial DNA polymerase [89], or in *SPG7* and *AFG3L2*, which encode for two subunits of the human mitochondrial inner membrane m-AAA protease [63]. However, in some cases, it is necessary to replace other parts of the human protein with that of yeast to allow complementation, creating a true chimeric polypeptide. This approach has been used for studying mutations in *OPA1*, which encodes for a mitochondrial dynamin like GTPase involved in mitochondrial fusion [128,210] and in *ANT1*, which encodes for a mitochondrial ADP/ATP carrier [174,211].

When the deletion of the yeast gene leads not only to an OXPHOS phenotype but also to the lethality or to the irreversible loss of mtDNA, other strategies can be used to create a relevant model. The most used is the plasmid shuffling strategy [212]. To this end, the gene is disrupted in a strain containing a plasmid with the selectable marker *URA3* and a wild-type copy of the gene. This strain is then transformed with a plasmid harbouring a different selectable marker and expressing the mutant allele. The treatment with 5-fluoroorotic acid (5-FOA) allows the growth of only those strains expressing hypomorphic mutant alleles. This is the case of some mutations in *GFER*, which encodes for a disulphide relay system protein [65]. When the deletion is associated to defects or loss of mtDNA, the selection on 5-FOA will allow to obtain cells containing only the mutant allele and assess the pathogenic role of the mutant variant. This approach has been used to study pathogenic mutations in *MIP1*, the yeast ortholog of *POLG* [213]. The advantage of this technique is that it is rapid, and the wild-type strain and the mutant ones are isogenic. A second strategy that can be used is based on the insertion of the mutant allele in a heterozygous diploid strain and performing tetrad analysis after sporulation. This approach has been used to study the effects of mutations in *MEF1* and *TUF1*, which encode for mitochondrial translation elongation factors [120]. A third strategy that can be used when gene deletion is associated to mtDNA loss relies on cytoduction, i.e., the fusion of the cytoplasm of the mutant strain devoid of mtDNA with the cytoplasm of a second strain [214]. This technique has been used for finding *MIP1* mutant alleles which behave as antimutators of the mtDNA [215]. However, these two last techniques have some limitations, mainly the non-isogenicity and the request of haploid strains with complementary auxotrophies, respectively.

## 3. Validation of Mutations and Understanding of Pathogenetic Mechanisms

### 3.1. Analysis of OXPHOS Phenotypes 

Once the model of the disease is obtained, the first, very quick way to validate (or not) an alleged pathological mutation is to test the ability of the mutant strain to grow in the presence of oxidative carbon sources such as glycerol, ethanol, acetate or lactate by a spot assay analysis. The growth of the mutant strain is then evaluated by comparison with the corresponding wild-type strain both at 28 °C, the optimal temperature for yeast growth, and at 37 °C, a temperature at which *S. cerevisiae* is still able to grow but often makes more easily evident the detrimental role of an amino acid substitution. 

Alternatively to the spot assay, a growth curve can be obtained to precisely and quantitatively evaluate the mutant growth performance, allowing the identification of minor defects not detectable with a semi-quantitative test such as a spot assay. Irrespective of the used test, if the strain carrying the mutant allele shows a complete absence or a reduction in oxidative growth, it is possible to conclude that the mutation is detrimental, thus validating it as pathological. In the first case, the mutation is very severe; in the second case, the mutation is leaky and it is possible to conclude that in this case the protein function is partially maintained. In contrast, if the mutant strain does not show a reduction in oxidative growth, it is not possible to exclude a causative role for the mutation tested and a deeper analysis must be carried out. Alternatively or in addition to an oxidative growth test, oxygen consumption rate (OCR) can be measured on whole cells grown in non-repressing conditions in which mitochondrial respiration is active. This analysis also allows to determine the severity of the damage, which in most cases correlates with the phenotypic severity in humans. 

Since one of the predominant roles of mitochondria is the production of energy through oxidative phosphorylation (OXPHOS), the enzymatic activity of the respiratory complexes and the rate of ATP synthesis could also be measured to compare mutants with the wild-type strains [216,217]. Additionally, mitochondrial membrane potential (ΔΨm) and the production of reactive oxygen species (ROS) could be evaluated because they are intimately connected to OXPHOS [218,219]. Moreover, to understanding exactly what is affected in the mutant strain, it is possible to explore a variety of phenotypic and molecular defects. The specific analyses appropriate to perform depend on the specific function of the protein encoded by the gene under investigation, as, for example, iron content measurement, mitochondrial dynamics or mitophagy [162,220].

A particular case of validation refers to a mutation found in a gene of unknown function and previously not associated with mitochondrial pathologies. This is, for example, the case of gene *LOC644096*, now termed *SDHAF1*, whose mutations lead to infantile leukoencephalopathy. The biochemical analysis of mitochondrial respiratory chain complexes performed in muscle and fibroblasts have shown a specific reduction in SDH and SCoQR. Because the transfection of fibroblasts with the gene *LOC644096* was not suitable to examine whether the disease-segregating missense mutations of *SDHAF1* were indeed causing cII deficiency, *S. cerevisiae* was used as a model. The putative *SDHAF1* yeast ortholog, *YDR379c-a*, an uncharacterised ORF of 239 bp was disrupted, and the null mutant resulted OXPHOS incompetent because of a profound and specific reduction in SDH activity. This suggested that *YDR379c-a*, named *SDH6,* encoded a protein which was specific for complex II. When yeast mutant alleles carrying the equivalent human mutations were created and introduced into the *sdh6Δ* mutant, the transformant strains behaved like the null mutant indicating the pathological effect of the mutations [45].

### 3.2. Determination of mtDNA Stability

Like its human counterpart, yeast contains several copies of mtDNA molecules, from 10–50 to 50–200 copies per cell, depending on the carbon source, growth temperature and haploid/diploid status [221,222]. As in human mitochondria, several copies of yeast mtDNA are packaged into 10–40 protein–DNA complexes, called nucleoids. These are anchored to the mitochondrial inner membrane [223,224,225] and contain proteins involved in packaging, replication, transcription, repair and recombination but also heat shock or Krebs cycle proteins [223,225,226,227,228,229].

One of the most peculiar characteristics of *S. cerevisiae* is *petite* positivity, i.e., it can survive without mtDNA. In this case, ATP is produced through alcoholic fermentation, provided that a fermentable carbon source is added in the medium, a condition resulting in colonies of small size called “*petite*”. The “*petite* phenotype” can be caused by mutations in nuclear genes (pet mutants) [230], or directly by mtDNA mutations (cytoplasmic *petite* mutants) [24]. Cytoplasmic *petite* mutants, called “*petites*”, arise spontaneously at high frequency even in a nuclear wild-type background (around 1–10% depending on the strain), and can be devoid of mtDNA (*rho*^0^ cells) or carry long deletions of mtDNA (*rho*^−^ cells); in the latter case, the mtDNA often contains several tandem repeats of the same sequences [231]. Cells containing whole mtDNA are called *rho*^+^. *Rho*^−^ mtDNA genomes are not very stable and may result in the loss of mtDNA, making the cell *rho*^0^. Mutations in several nuclear genes involved in the replication, recombination and repair of the mtDNA, but also in its maintenance and integrity, can affect the *rho* status of the cells (reviewed in [232]).

When a mutation in a mtDNA molecule occurs, the cell is heteroplasmic. Contrary to what happens in mammals, heteroplasmy is just a transient condition in yeast, giving rise, in a few generations, to two homoplasmic populations of cells, each with only a kind of mtDNA genome [233,234,235]. The effects of nuclear mutation on mtDNA stability can be measured through the determination of the *petite* frequency, i.e., the ratio between the number of *petites* colonies and the number of total colonies. Although yeast cells are homoplasmic, and human cells are mainly heteroplasmic, a population of yeast cells recapitulates the heteroplasmic status of a single human cell. The higher the frequency of *petites* is, the higher the detrimental effect of the mutation is on the maintenance of the integrity and on stability of the mtDNA. It must be underlined that the *petite* frequency depends on two factors: an intrinsic factor, which depends on both the strain background, in particular the nuclear mutation under investigation, which influences the onset of *petites* per generation, and on the growth rate of *rho*^+^ vs. *petite* cells, which is generally different depending on the strain; an extrinsic factor which depends on the growth conditions, such as the medium, carbon source, and temperature, which can influence both the onset of *petite* cells and the growth rates [232]. Then, it is critical that the comparison between mutant and wild type is performed in the same genetic background and in the same growth conditions. All the methods used to measure the *petite* frequency, described in [212], are based on a pre-growth in an oxidative carbon source to minimise the presence of *petites.* This is followed by growth in a medium supplemented with a fermentable carbon source, such as glucose, for several generations (at least 10–15), to allow the onset and the growth of *petite* cells. This analysis can be conducted on a cell population or on cells deriving from single colonies. The first method offers the advantage that the onset of *petites* occurs independently several times in several cells, resulting in a frequency that is rather constant, whereas in the second case, the *petite* frequency of each colony is highly variable since the number of *petites* is strongly influenced by the time of the onset of the first *petite* cell and thus the results must be analysed as in a fluctuation test based on the median. 

Moreover, to discriminate between *rho*^−^ and *rho*^0^ cells, and then to distinguish if a nuclear mutation results primarily in deletions or in depletion of mtDNA, three main methods can be applied [212]: (i) crossing a number of *petite* cells with different *mit*^−^ tester strains, harbouring a single point mutation in a mitochondrial gene encoding for a respiratory complex subunit; if at least one of the diploids obtained is respiratory proficient, it means that the tested cell retained a mtDNA fragment encompassing the *mit*^−^ mutation and then it was *rho*^−^; (ii) analysis by the Southern Blot of the mtDNA extracted from *petite* colonies using an *ori* fragment as a probe; (iii) staining of mtDNA with DAPI (4′,6-Diamidine-2′-phenylindole dihydrochloride), which, in yeast, binds both the nuclear DNA and the mtDNA. By these techniques, it was shown that some mutations in *POLG/MIP1* increase primarily the frequency of *rho*^−^ colonies, whereas others mostly increase the frequency of *rho*^0^ colonies [77,78].

Some nuclear mutations also result in a decrease in the number of intact mtDNA molecules, i.e., the cells are respiratory proficient, but contain less mtDNA. The relative mtDNA levels can be measured through qPCR, amplifying a region of one of the mitochondrial protein genes as the target and a region of the nuclear DNA, such as *ACT1*, as the control [236]. By comparing the mtDNA/nuclear DNA ratio of a nuclear mutant strain and of the corresponding wild-type strain, it is possible to evaluate whether the mutation is associated to the depletion of mtDNA. An example of this approach is the study of the effects of polymorphisms/mutations in *POLG/MIP1* on the mtDNA [237]. 

Mitochondrial fusion and fission have a critical role in several aspects of the mitochondrial metabolism, among which are the replication and fidelity of the mtDNA [238,239]. Indeed, the absence of fusion results in the complete loss of mtDNA in yeast [240,241] and the partial loss of mtDNA in mammalian cells [242]. Mitochondrial fission prevents the clustering of nucleoids resulting in an unbalanced distribution of mtDNA copies within the mitochondria. Interesting enough, concomitantly inhibiting fission and fusion can suppress cellular dysfunction, including in yeast and in human cells [243]. Due to the evolutive conservation of genes also associated to fission and fusion, the consequences of human mutations have been evaluated in yeast. As an example, expression of a the *MGM1-OPA1* chimeric construct was used to model both dominant and recessive human pathological mutations in *OPA1,* associated to optic atrophy (DOA), to DOA^+^ and to pathologies associated to mtDNA depletion [127,128]. However, in mammalian cells, mitochondrial fusion, allowing mtDNA genomes with distinct mutations to complement each other, seems to ameliorate the detrimental effects of heteroplasmic mtDNA mutations and then the clinical severity of inherited mtDNA encephalomyopathies [242].

### 3.3. Analysis of Mitochondrial Protein Synthesis (MPS)

A correct OXPHOS metabolism may also depend on the correct gene expression and translation of the mtDNA. In particular, mutations in all the nuclear-encoded proteins involved in mitochondrial transcription, the processing and maturation of the RNAs, the aminoacylation of the tRNA and translation can result in defects of the MPS and, in turn, in defects of the OXPHOS system. In addition to specific analyses linked to specific genes under investigation, such as the determination of the maturation of the RNA, the presence of specific modifications in the tRNAs and the levels of aminoacylated tRNA, [100,103,244,245], the main analysis used to evaluate such defects is the measurement of the MPS. 

The two main methods for measuring the MPS are based on an SDS-PAGE gel electrophoresis of proteins labelled in vivo or in organello [246]. In both cases, cells are grown in medium supplemented with a non-fermentable carbon source, if the strain is respiratory proficient, or in non-repressing conditions, such as with galactose, or with glucose at low concentrations until exhaustion. The presence/absence and the quantity of the eight mitochondrial-encoded proteins (in order, the mitochondrial subunit Var1, and the OXPHOS complexes subunits Cox1, Cox2, Cob, Cox3, Atp6, Atp8/Atp9) can thus be assessed and the comparison between the mutant strain and the corresponding wild-type allows the identification of those mutations which affect the protein synthesis. Mutations in some genes, such as those encoding for aminoacyl-tRNA synthetase, generally reduced the levels of all the mitochondrial proteins, since the whole protein synthesis is compromised. Mutations in other genes, on the contrary, mostly or specifically affect the synthesis of specific proteins. For example, mutations equivalent to the human ones in *MTO1*, which encodes for a subunit of the complex which catalyses the 5-carboxymethylaminomethyl modification of the wobble uridine base in mitochondrial tRNAs, affected specifically the synthesis of Cox1, Cox2 and Cob; on the contrary, mutations in *TRZ1*, which encodes for the tRNA 3’-end processing endonuclease tRNase Z, affected the synthesis of all the mitochondrial proteins, though at a different extent [92,95]. 

### 3.4. Evaluation of Protein Stability 

Another aspect that can be evaluated in the yeast models of diseases is the effect of missense mutations on the stability/quantity of the protein. A reduction in the protein steady-state level could be the molecular cause of the disease or at least a major contributor. Unfortunately, only a few commercial antibodies are available to date that specifically recognise yeast mitochondrial proteins. To overcome this limitation, the addition of a polypeptide tag enables revealing the protein under analysis with an antibody against the tag sequence. However, it is necessary to exclude that the tag addition interferes with protein import and protein function [70]. 

To assess the functionality of the fusion protein, a complementation test to evaluate oxidative growth is performed comparing the phenotype of the strain expressing the tagged vs. untagged wild-type protein. The steady-state level of mutated proteins is then analysed by Western blot and immunodetection, directly on whole cell protein extract and using cytosolic or mitochondrial markers as the loading control. Interestingly, the overexposure of the signals could allow to evidence a degradation product pointing out that the mutant protein is unstable. Such a situation was, for example, observed by studying the human mutation R183Q in the pitrilysin metallopeptidase 1 encoded by *PITRM1* in yeast, taking advantage of the presence of the orthologous gene, *CYM1*. This autosomal recessive missense mutation, associated with protein instability, was found in two patients presenting a slowly progressive neurodegenerative disease characterised by mental retardation, spinocerebellar ataxia, cognitive decline and psychosis [68].

### 3.5. Analysis of Mutant Proteins Localisation

The pathogenic variant can also interfere with the correct protein localisation into mitochondria. To assess this possibility and take advantage of the available antibody or a tag antibody, Western blot analysis could be performed. This approach was used to analyse mutations in *MPV17*, associated to the hepatocerebral form of mitochondrial DNA depletion syndromes (MDDS) and Navajo neuro-hepatopathy, using the yeast orthologous gene *SYM1*. Both genes encode for a small protein localised to the inner mitochondrial membrane, whose function is not yet fully understood. The impact of seven pathological missense mutations, localised in different protein domains, on correct mitochondrial localisation was assessed demonstrating that the mutated residues do not compromise protein import [75]. 

By exploiting yeast strains expressing pathogenic variants, it is also possible to evaluate if they interfere with the ability of the mutated protein to be part of a complex. A relevant fraction of mitochondrial proteins is in fact localised in the inner mitochondrial membrane [70] and some of these are organised into complexes. This is, for example, the case for the proteins of the electron transport chain complexes, except for complex I that is not present in *S. cerevisiae*, where it is replaced by a non-proton-translocating NADH dehydrogenase activity performed by a single protein: Ndi1p [247]. Blue native polyacrylamide gel electrophoresis (BN-PAGE) technique, initially set up to study principally mitochondrial respiratory chain enzymes [248], can be used to analyse any protein complex [249] as respirasomes (supercomplexes derived by different stoichiometric aggregates of the respiratory complexes) [250]. For example, this technique was used to assess the effects of pathogenic mutations in the cIV assembly factor SURF1, Shy1 in yeast, demonstrating that pathological variants compromise the cIV assembly and the formation of the supercomplexes with the cytochrome bc1 complex [55,56]. With the same technique, it was demonstrated that Sym1, the equivalent of MPV17, takes part in a high molecular–weight complex of which the composition is still unknown [251]. Furthermore, the impact of seven *MPV17/SYM1* missense mutations was assessed showing that six of them compromised the formation of the fully assembled complex [75]. 

## 4. Inheritance Pattern Analysis: Dominance/Recessivity and Gene Interactions Analysis

A great advantage offered by yeast is the possibility to have information on the dominance/recessivity of mutations, which is not always easy to collect in patients, especially in sporadic cases with no familial history. The dominance/recessivity of a mutation can be established by comparing the phenotype of a diploid heterozygous strain harbouring the mutation under analysis with an isogenic diploid homozygous strain. If the phenotype of the heterozygous strain is similar to that of the homozygous strain, this means that the mutation is recessive, although it is not possible to discriminate whether the allele is null or hypomorphic and, in the latter case, the degree of hypomorphism. On the contrary, if the heterozygous strain shows a detrimental phenotype compared with the homozygous strain, it means that the human mutation is dominant, resulting in an antimorphic allele or in a neomorphic allele and causing a negative dominance or a gain-of-function dominance, respectively. However, the dominance can also be due to haploinsufficiency. 

Several yeast genes, when disrupted in a single copy in a diploid strain, cause haploinsufficiency (www.yeastgenome.org, [252,253]). For genes encoding mitochondrial proteins, haploinsufficiency, if present, generally results in a decrease in respiratory growth, of respiratory activity and/or of mtDNA stability, i.e., the hemizygous shows a detrimental phenotype compared to the wild-type homozygous strain.

If haploinsufficiency occurs for the gene under investigation, more detailed information can be inferred by comparing the diploid heterozygous with both a wild-type diploid homozygous and the hemizygous strain. Depending on the heterozygous strain genetic background, the hemizygous can be a diploid deleted in one gene copy or a null strain transformed with two plasmids, one empty and the other harbouring the wild-type allele.

When the heterozygous strain shows a phenotype similar to that of the hemizygous one or intermediate between the homozygous strain and hemizygous one, it means that the allele is null or hypomorphic, respectively, and the mutation causes a dominant pathology due to haploinsufficiency. However, if in humans no haploinsufficiency is associated to the gene under analysis, the mutation will likely behave as recessive and the pathology occurs only when in homozygosis or in compound heterozygosis with a second mutation. When the phenotype of the heterozygous strain is more detrimental than that of the hemizygous strain, it suggests that the human mutation alone can be the cause of the pathology due to a negative dominance or to a gain-of-function dominance.

On the assumption that the hemizygous strain shows haploinsufficiency, when heterozygous and homozygous strains show similar phenotypes, the mutation is recessive; this means that the allele is hypomorphic and the human mutation is pathological in compound with a second mutation or is a phenotypic modifier. 

An example of this genetic analysis is the study on mutations in *MIP1*, the ortholog of the human *POLG* (reviewed in [213]). Some mutations in the polymerase domain abolish the maintenance of the mtDNA in the haploid strain, whereas the heterozygous strain shows an increase in the *petite* frequency compared to the hemizygous strain: these mutations cause dominant pathologies in patients. Instead, other mutations strongly increase the *petite* frequency or make the haploid strain *rho*^0^, whereas the behaviour of the heterozygous is intermediate or similar to that of the hemizygous strain. Considering that *POLG* does not show haploinsufficiency in humans, these mutations are typically recessive and are found in homozygosis or in compound heterozygosis in patients. Regarding *MIP1*, it should also be noted that some mutations have a slight effect in the haploid background and any effect when compared with heterozygous and wild-type homozygous strains. These mutations are typically polymorphisms which behave as phenotypic modifiers worsening the effects of the pathological mutations in compounds. Another example of dominance/recessive analysis concerns the gene *ISCU*, whose model has been shown in paragraph 2, and whose recessive mutations have been associated to diseases in humans. The analysis performed in yeast allowed the identification of the first dominant mutation, as highlighted by the fact that the heterozygous strain was associated with a respiratory deficient phenotype [162].

Yeast can also be useful when more mutations are present in the patients, in order to understand which mutations are responsible for the pathology and if an additive/synergistic effect occurs, suggesting a functional interaction of the mutated amino acids. Indeed, some patients show two mutations in cis and/or in trans. This is rather common for genes in which several SNPs/mutations are present in the population, especially those which encodes for long mRNA such as *OPA1* (https://databases.lovd.nl/shared/genes/OPA1 (accessed on 18 February 2021)) [254] or POLG (https://tools.niehs.nih.gov/polg/ (accessed on 18 February 2021)) [255], in which more than 250 pathological variants have been identified. If two mutations are present in cis, the role of each mutation can be dissected in yeast by introducing the single mutation in the gene under analysis. In this case, three mutant alleles are constructed: an allele containing one of the two mutations, an allele with the other mutation and the double mutant allele. By comparing the phenotype of each haploid strain harbouring these mutant alleles with the wild-type haploid strain, four main cases are possible. First, both single mutant alleles are neutral, but the double mutant allele is associated to a detrimental phenotype: in this case the mutations are not pathological alone, but just in compound, suggesting a functional interaction. Second, a single mutant allele is associated to a detrimental phenotype and the double mutant allele has the same behaviour: one mutation is pathological whereas the other is a neutral SNP. Third, a single mutant allele is associated to a detrimental phenotype, and the double mutant allele leads to a worse phenotype: one mutation is pathological whereas the other is a phenotypic modifier which can influence the phenotype of the pathological mutation. Fourth, both single mutant alleles are associated to a detrimental phenotype, and in the double mutant, the phenotypic effects are additive or synergistic: both mutations are pathological alone, and when in compound, negatively affect the effect of the other one. Examples of such analyses, with different outcomes, have been performed for *MIP1* and *DNM1*, which encodes for a dynamin-related GTPase involved in mitochondrial organisation [78,124].

If two mutations are present in trans, suggestive of a recessive pathology, the analysis to distinguish the role of each mutation can be performed by comparing the heterozygous diploid strain harbouring both mutant alleles with the wild-type homozygous strain, and with two heterozygous strains each harbouring a single mutant allele and a wild-type allele, and with two homozygous mutants each harbouring one of the two mutations in both alleles. If the mutations were both recessive, it is expected that the compound heterozygous strain has a detrimental phenotype compared to the homozygous wild type as well as to the single heterozygous ones. However, other information can be inferred by the comparison with both the single heterozygous and the homozygous mutant strains. For example, thank to this comparison, we demonstrated that a mutation in *MIP1* found in compound heterozygosis with a second mutation was dominant, and acted synergistically with a second, recessive mutation in trans [78].

## 5. Yeast as A Model for Mitochondrial Diseases Drug Discovery 

To date, no effective treatments exist for mitochondrial diseases [256]. In recent years, phenotype-based screenings have been proposed in yeast models to find drugs able to suppress OXPHOS phenotypes associated with mitochondrial diseases mutations. 

High throughput drug-screening (HTS) was performed in the case of Friedreich’s ataxia (FRDA), caused by mutations in the nuclear gene *FXN* that encodes the highly conserved frataxin, a chaperone for iron-sulphur cluster (ISC) assembly in the mitochondrial matrix [257]. More than 100,000 compounds were screened for their ability to improve mitochondrial functions in yeast lacking the expression of *YFH1* gene, the functional orthologous of *FXN*. The rescue was recorded by a colorimetric assay, quantitatively monitoring cell metabolic activity on respiratory substrates [258]. 

Alternatively, a phenotype-based screening, named “drug drop test” [20,259] has been developed, in which yeast mutants, defective in oxidative growth due to the alteration of mitochondrial functionality, are initially spread on a solid medium. The mutants are then exposed to compounds from chemical libraries, spotted on small sterile filters placed on the agar surface. The appearance of a halo of enhanced growth around a filter indicates the effect of the corresponding drug. The strength of this method is that the diffusion of the molecule around the filter in the agar medium creates a concentration gradient, making it unnecessary to find the optimal drug concentration by testing different dilution of the compound. The screen allows to rapidly analyse chemical libraries, like those containing FDA-approved drugs (i.e., Prestwick or Selleck), in a repurposing approach. The drug drop test was used for the first time to identify drugs active in the yeast model for ATP synthase disorders, associated with neurodegenerative syndromes including neuropathy, ataxia and retinitis pigmentosa (NARP) [259]. Active compounds were then confirmed as effective in cybrid-based model of NARP.

Phenotype-based screenings enable the identification of potential therapeutic compounds in the absence of validated drug targets and independently on the knowledge of their mechanism of action. Yeast offers additional experimental tools, like the collection of homozygous and heterozygous deletant mutants, by which chemical–genomic experiments can be performed obtain an indication on the molecular mechanism of active drugs. In the haploinsufficiency profile (HIP) approach, the reduced fitness of a heterozygous deletion mutant to the inhibitory concentration of a drug indicates that the deleted gene is the molecular target of the drug [260]. By a chemical genomic analysis of haploinsufficiency, heterozygous Tim17 or Tim23, the components of the translocase inner mitochondrial membrane involved in mitochondrial protein import, displayed sensitivity to sodium pyrithione (NaPT) [261]. In vitro experiments indicated that NaPT specifically influences the import of pre-sequence proteins via Tim23 complex and indicates the machinery of the mitochondrial import as a potential target for a therapeutic approach.

By the drug drop test, two molecules, the antibiotic pentamidine and clarithromycin, have been found to actively restore oxidative growth and OXPHOS phenotypes due to mutations in the yeast *BCS1* gene, the orthologue of the human *BCS1L* [262]. It has been demonstrated that pentamidine and clarithromycin target mitochondrial rRNA, thus altering the synthesis of mitochondrial encoded OXPHOS subunits, with the consequent alteration of the OXPHOS complex assembly. However, the two antibiotics were able to rescue the respiratory phenotypes of *bcs1* mutants carrying missense mutations, that not completely compromise the Bcs1 activity, but did not restore phenotypes due to point mutations or deletion that fully abolish Bcs1 function. In vivo experiments performed in yeast also allowed to propose a model of action of the two drugs. 

Very recently, taking advantage of the impaired oxidative growth of a strain carrying a mutation in the *CAB1* gene, the ortholog of the human *PANK2* that encodes the panthotenate kinase (PANK), a screening of the Selleck chemical library has been performed. Two molecules in particular, nalidixic acid and 5,7 dichloro-8-hydroxyquinoline, were found to be able to restore the multiple defects associated with PANK deficiency, with the rescue not being allele-specific [169].

Yeast-based screenings have also been used to determine therapeutic strategies against mitochondrial diseases affecting mtDNA stability. This is the case of DOA, and of the more severe form named DOA-plus, the most common mitochondrial optic neuropathies, characterised by the gradual loss of vision as a result of the degeneration of the optic nerve cells. These pathologies are mainly caused by mutations in the nuclear gene *OPA1*, encoding a mitochondrial GTPase implicated in mtDNA maintenance, [263,264,265] whose functional orthologue in yeast is *MGM1* gene [266]. Drugs were first selected as able to restore the thermal sensitive (ts) growth on respiratory substrates of a yeast mutant harboring *mgm1^I322M^* mutation. This mutation is equivalent to I382M mutation in *OPA1* [127], one of the few pathological mutations that can be modelled in yeast, due to the low similarity between *MGM1* and *OPA1* sequences. Positive hits were then subject to a subsequent screening, using a strain harbouring the ts *chim3^S646L^* mutation in the *MGM1/OPA1* chimeric allele, encoding the N-terminal region of Mgm1 and the whole GTPase, middle and GED domains of OPA1 [128] (see paragraph 2) identifying six effective drugs. Five of them also ameliorated, to a different extent, the pathological OXPHOS phenotypes of *Opa1* null mouse embryo fibroblasts (MEFs), that express the human OPA1 isoform 1, bearing R445H and D603H mutations, associated with DOA-plus and DOA, respectively. The analysis in patient’s fibroblasts bearing the same mutations allowed to identify the tolfenamic acid, a non-steroidal anti-inflammatory drug, as the most promising therapeutic compound and to propose the repurposing of this drug in a clinical trial for neurodegenerative diseases associated with *OPA1* mutations [267]. 

Therapeutic molecules were also found via yeast-based screening for a broad spectrum of mitochondrial pathologies, including Alper syndrome, ataxia neuropathy, dominant and recessive progressive external opthalmoplegia (arPEO and adPEO), characterised by mtDNA deletions or depletion consequent to the mutation in the mtDNA polymerase POLG [255]. 

Two chemical libraries of FDA-approved molecules were screened, taking advantage of one of these *mip1* mutant (G651S equivalent to POLG G848S) whose ts mutation conferred an evident but not irreversible phenotype. The clofilim tosylate (CLO), belonging to a class of anti-arrhythmic agents, displayed the best rescuing activity, suppressing the respiratory growth defect and preventing mtDNA loss in all *mip1* mutants tested [268]. The rescuing effect of CLO was later validated in two animal disease models, *C. elegans* and zebrafish [269], as well as in the fibroblasts of a patient carrying compound heterozygous *POLG* mutations. The molecular mechanism by which CLO exerts the rescuing activity is not yet known, however, the successful application in this four-model approach indicates that CLO is acting by a rescuing mechanism conserved through the evolution. 

## 6. Conclusions

Early studies and more recently, NGS techniques, have made possible the identification of a huge number of novel human genetic variants whose causality in determining mitochondrial syndromes was demonstrated through functional analyses, often in model systems. Because of the evolutionary conservation of genes and systems, the study of human genetic defects associated with mitochondrial dysfunction has often been directly addressed in the model organism *Saccharomyces cerevisiae,* the molecular and genetic workhorse for much of our understanding of mitochondrial biogenesis in eukaryotes. As shown in Table 1, *S. cerevisiae* helped to resolve the cause of OXPHOS diseases for about a third of the disease genes known today. It can also be noted that the contribution to the identification of causative genes-concerned variants associated to genes with a wide range of mitochondrial functions, from those that have a specific role in OXPHOS biogenesis, as defects in respiratory complexes or in DNA maintenance and expression, to those that have a secondary impact on OXPHOS caused by deficiency in protein import and processing, metabolite and electron transport, membrane dynamics and composition, TCA cycle and metabolism, Fe–S cluster biogenesis and protein quality control. 

Today, one of the major challenges in the field of mitochondrial diseases is the identification of the genetic basis of the conditions of patients lacking a diagnosis, generally because of the clinical and genetic heterogeneity of these pathologies. Certainly, international collaboration within the mitochondrial disease field will improve the identification of additional cases with similar clinical phenotypes and above all, of new disease genes. Model systems will therefore continue to prove being fundamental in proving the pathogenicity of the variants and in improving our understanding of the role played by different genes. In this context, yeast remains a powerful model for discovering the function of mitochondrial proteins, especially for those yet to be characterised. In fact, it should be remembered that many proteins present in the mitochondrial proteome still lack any detailed characterisation. Furthermore, another aspect still minimally considered is the indirect effect of cytoplasmic proteins on mitochondrial biogenesis. A screening recently done in yeast, on the negative effect of heat stress on respiratory capacity, allowed to expand the repertoire of genes affecting mitochondrial function, allowing the identification of 105 new genes and novel pathways, whose corresponding proteins are predominantly present in the cytoplasmic proteome [270]. 

Another major challenge relative to the mitochondrial disease is their treatment. Their heterogeneity, the intrinsic variability of mitochondrial genetics, the fact that they directly or indirectly affect several organs via ATP production, and the impossibility for many drugs to reach the brain, which is often affected in mitochondrial disorders, represent the main problems in the development of effective therapies [256]. A radical cure for these diseases will probably only come from gene therapy. However while “tailored”, personalised therapeutic approaches, such as gene therapy, cell therapy and organ replacement can be useful for individual conditions [256], their costs cannot be easily supported by health authorities, given the large number of patients affected by mitochondrial diseases. For this reason, the identification of therapeutic molecules effective on a broader spectrum of mitochondrial pathologies remains an important objective to alleviate, if not eliminate, the defects responsible for the diseases. In this context, given the long times required for drug development and the numerous and different targets of drugs, repurposing available therapeutic molecules remains an interesting way forward. Even from this point of view, yeast disease models have demonstrated to be useful, thanks to the techniques here reported, allowing to quickly analyse a large number of molecules. Some of these compounds were found to be effective also in the corresponding animal models and in patients fibroblasts, despite the more complex genetic interactions present in animals, including humans, than in yeast. The numerous disease models available in yeast can also be explored to identify beneficial broad-spectrum molecules.

In addition to the great advantages of the yeast model, it should be noted that some limitations exist: (i) yeast cannot be used to model a disease at the scale of an organ or an intact complex multicellular organism; (ii) yeast does not allow to assess tissue specificity and disease progression; (iii) some functions fulfilled by human mitochondria do not exist in *S. cerevisiae* such as the respiratory complex I which, however, is present in the yeast *Yarrowia lipolytica*, used to model mutations related to this complex [271]. Moreover, cell division and mitochondrial replication in human development could lead to much greater variation in the relative levels of the mtDNA mutation in a largely stochastic system [23]. This could induce compensatory biogenesis mechanisms to maintain the cell’s mitochondrial function, an effect not observable in yeast where the heteroplasmic condition is rapidly lost. Despite these limitations, mitochondrial function conservation between yeast and humans renders yeast a key model for mitochondrial medicine.

## Figures and Tables

**Table 1:** List of genes linked to mitochondrial diseases categorised according to their primary role. This table includes nuclear genes associated to mitochondrial disorders in humans with an orthologous gene in *S. cerevisiae* * for whom the yeast model was reported in the literature to have been used to validate the pathogenic role of the variants associated with disease.

Function		Human/Yeast Gene	References *
OXPHOS subunits	CII	*SDHA/SDH1*	[38]
		*SDHB/SDH2*	[39,40]
		*SDHD/SDH4*	[41,42]
	CIV	*COX6B1/COX12*	[43]
	CV	*ATP5E/ATP15*	[44]
OXPHOS assembly factors	CII	*SDHAF1/SDH6*	[45]
	CIII	*BCS1L/BCS1*	[46,47,48,49,50]
		*LYRM7/MZM1*	[51,52]
	CIV	*COX10/COX10*	[53,54]
		*SURF1/SHY1*	[55,56]
	CV	*ATPAF2/ATP12*	[57,58]
Protein import and processing		*AFG3L2/AFG3*	[59,60,61,62,63,64]
		*GFER/ERV1*	[65,66]
		*MIPEP/OCT1*	[67]
		*PITRM1/CYM1*	[68,69]
		*PMPCB/MAS1*	[70]
		*SPG7/YTA12*	[60,62,63]
		*TIMM50/TIM50*	[71]
		*TIMM8A/TIM8*	[72,73]
mtDNA replication, transcription and maintenance		*MPV17/SYM1*	[74,75]
		*POLG/MIP1*	[76,77,78,79,80,81,82,83,84,85,86,87,88,89,90]
		*POLR2A/RPB1*	[91]
RNA maturation/modification		*ELAC2/TRZ1*	[92]
		*GTPBP3/MSS1*	[93]
		*MRM2/MRM2*	[94]
		*MTO1/MTO1*	[95,96]
		*TRIT1/MOD5*	[97]
		*TRMT5/TRM5*	[98]
		*TRMU/MTO2*	[99,100]
		*TRNT1/CCA1*	[101,102]
Mitochondrial aminoacyl tRNA synthetases		*AARS2*/*ALA1*	[103,104]
		*GARS/GRS1*	[105,106,107]
		*GATB/PET112*	[108]
		*HARS2/HTS1*	[109]
		*KARS/MSK1*	[110]
		*LARS2/NAM2*	[111]
		*QRSL1/* *HERS2*	[108]
		*RARS2/MSR1*	[112]
		*TARS2/MST1-THS1*	[113]
		*VARS2/VAS1*	[114,115]
		*WARS2/MSW1*	[116]
		*YARS2/MSY1*	[117,118,119]
Translation		*GFM1/MEF1*	[120]
		*TUFM/TUF1*	[120,121,122]
Membrane dynamics and composition		*DNM1L/DNM1*	[123,124,125]
		*MFN2/FZO1*	[126]
		*OPA1/MGM1*	[127,128]
		*TAZ/TAZ1*	[129,130,131]
		*VPS13C/VPS13*	[132,133,134]
		*APOO/MIC26 paralog MIC27*	[135]
		*GDAP1/*	[136]
		*ACO2/ACO1*	[137,138,139,140]
		*IDH3A/IDH2*	[141]
		*MDH2/MDH1*	[142]
		*MECR/ETR1*	[143]
		*MPC1/MPC1*	[144]
		*PDHA1/PDA1*	[145]
		*PDHX/PDX1*	[146]
		*PPA2/PPA2*	[147]
		*SLC25A13/AGC1*	[148]
		*SLC25A3/PIC2-MIR1*	[149,150]
Fe–S cluster biogenesis		*ABCB7/ATM1*	[151,152,153]
		*FDXR/ARH1*	[154]
		*FXN/YFH1*	[155,156,157,158,159,160]
		*ISCU/ISU1 paralog ISU2*	[161,162]
		*LYRM4/ISD11*	[163]
		*NFU1/NFU1*	[164]
Enzyme co-factors		*COASY/CAB5*	[165,166]
		*LIPT1/LIP3*	[167]
		*LIPT2/LIP2*	[168]
		*PANK2/CAB1*	[169]
Metabolite transport		*SLC25A1/CTP1*	[170]
		*SLC25A4/AAC2*	[171,172,173,174,175,176,177,178,179]
		*SLC25A19/TPC1*	[180]
		*SLC25A32/FLX1*	[181]
Electron carriers	CoQ	*COA6/COA6*	[182,183]
		*COQ2/COQ2*	[184,185,186,187]
		*COQ4/COQ4*	[188,189]
		*COQ5/COQ5*	[190]
		*COQ6/COQ6*	[191,192]
		*COQ8A/COQ8*	[193]
		*COQ8B/COQ8*	[194]
		*COQ9/COQ9*	[195,196]
		*PDSS1/COQ1*	[187]
	Cytc	*CYCS/CYC1 paralog CYC7*	[197,198]
		*HCCS/CYC3*	[199,200,201]

## Data Availability

Data sharing not applicable.

## References

[1] Thompson K., Collier J.J., Glasgow R.I.C., Robertson F.M., Pyle A., Blakely E.L., Alston C.L., Oláhová M., McFarland R., Taylor R.W. (2020). Recent Advances in Understanding the Molecular Genetic Basis of Mitochondrial Disease. J. Inherit. Metab. Dis..

[2] Zeviani M., Di Donato S. (2004). Mitochondrial Disorders. Brain.

[3] Rustin P., Bourgeron T., Parfait B., Chretien D., Munnich A., Rötig A. (1997). Inborn Errors of the Krebs Cycle: A Group of Unusual Mitochondrial Diseases in Human. Biochim. Biophys. Acta.

[4] Hiltunen J.K., Schonauer M.S., Autio K.J., Mittelmeier T.M., Kastaniotis A.J., Dieckmann C.L. (2009). Mitochondrial Fatty Acid Synthesis Type II: More than Just Fatty Acids. J. Biol. Chem..

[5] Herst P.M., Rowe M.R., Carson G.M., Berridge M.V. (2017). Functional Mitochondria in Health and Disease. Front. Endocrinol..

[6] Rawat S., Stemmler T.L. (2011). Key Players and Their Role during Mitochondrial Iron-Sulfur Cluster Biosynthesis. Chemistry.

[7] Letai A.G. (2008). Diagnosing and Exploiting Cancer’s Addiction to Blocks in Apoptosis. Nat. Rev. Cancer.

[8] Rimessi A., Giorgi C., Pinton P., Rizzuto R. (2008). The Versatility of Mitochondrial Calcium Signals: From Stimulation of Cell Metabolism to Induction of Cell Death. Biochim. Biophys. Acta.

[9] Dimauro S., Davidzon G. (2005). Mitochondrial DNA and Disease. Ann. Med..

[10] Zeviani M., Carelli V. (2007). Mitochondrial Disorders. Curr. Opin. Neurol..

[11] Suomalainen A., Isohanni P. (2010). Mitochondrial DNA Depletion Syndromes--Many Genes, Common Mechanisms. Neuromuscul. Disord..

[12] Vafai S.B., Mootha V.K. (2012). Mitochondrial Disorders as Windows into an Ancient Organelle. Nature.

[13] Schaefer A.M., Taylor R.W., Turnbull D.M., Chinnery P.F. (2004). The Epidemiology of Mitochondrial Disorders--Past, Present and Future. Biochim. Biophys. Acta.

[14] Mancuso C., Scapagini G., Currò D., Giuffrida Stella A.M., De Marco C., Butterfield D.A., Calabrese V. (2007). Mitochondrial Dysfunction, Free Radical Generation and Cellular Stress Response in Neurodegenerative Disorders. Front. Biosci..

[15] Ng Y.S., Turnbull D.M. (2016). Mitochondrial Disease: Genetics and Management. J. Neurol..

[16] Niyazov D.M., Kahler S.G., Frye R.E. (2016). Primary Mitochondrial Disease and Secondary Mitochondrial Dysfunction: Importance of Distinction for Diagnosis and Treatment. Mol. Syndromol..

[17] Keogh M.J., Chinnery P.F. (2015). Mitochondrial DNA Mutations in Neurodegeneration. Biochim. Biophys. Acta.

[18] Ahuja P., Wanagat J., Wang Z., Wang Y., Liem D.A., Ping P., Antoshechkin I.A., Margulies K.B., Maclellan W.R. (2013). Divergent Mitochondrial Biogenesis Responses in Human Cardiomyopathy. Circulation.

[19] Stenton S.L., Prokisch H. (2018). Advancing Genomic Approaches to the Molecular Diagnosis of Mitochondrial Disease. Essays Biochem..

[20] Lasserre J.-P., Dautant A., Aiyar R.S., Kucharczyk R., Glatigny A., Tribouillard-Tanvier D., Rytka J., Blondel M., Skoczen N., Reynier P. (2015). Yeast as a System for Modeling Mitochondrial Disease Mechanisms and Discovering Therapies. Dis. Model. Mech..

[21] Pagliarini D.J., Calvo S.E., Chang B., Sheth S.A., Vafai S.B., Ong S.-E., Walford G.A., Sugiana C., Boneh A., Chen W.K. (2008). A Mitochondrial Protein Compendium Elucidates Complex I Disease Biology. Cell.

[22] Calvo S.E., Clauser K.R., Mootha V.K. (2016). MitoCarta2.0: An Updated Inventory of Mammalian Mitochondrial Proteins. Nucleic Acids Res..

[23] Stewart J.B. (2020). Current Progress with Mammalian Models of Mitochondrial DNA Disease. J. Inherit. Metab. Dis..

[24] Ephrussi B., Slonimski P.P. (1955). Subcellular Units Involved in the Synthesis of Respiratory Enzymes in Yeast. Nature.

[25] Schatz G., Haslbrunner E., Tuppy H. (1964). Deoxyribonucleic acid associated with yeast mitochondria. Biochem. Biophys Res. Commun..

[26] Winzeler E.A., Shoemaker D.D., Astromoff A., Liang H., Anderson K., Andre B., Bangham R., Benito R., Boeke J.D., Bussey H. (1999). Functional Characterization of the S. Cerevisiae Genome by Gene Deletion and Parallel Analysis. Science.

[27] Guthrie C., Fink G. (1991). Guide to Yeast Genetics and Molecular Biology. Methods in Enzymology.

[28] Guthrie C., Fink G. (2002). Guide to Yeast Genetics and Molecular and Cell Biology—Part B. Methods in Enzymology.

[29] Foury F. (1997). Human Genetic Diseases: A Cross-Talk between Man and Yeast. Gene.

[30] Foury F., Kucej M. (2002). Yeast Mitochondrial Biogenesis: A Model System for Humans?. Curr. Opin. Chem. Biol..

[31] Prokisch H., Scharfe C., Camp D.G., Xiao W., David L., Andreoli C., Monroe M.E., Moore R.J., Gritsenko M.A., Kozany C. (2004). Integrative Analysis of the Mitochondrial Proteome in Yeast. PLoS Biol..

[32] Reinders J., Zahedi R.P., Pfanner N., Meisinger C., Sickmann A. (2006). Toward the Complete Yeast Mitochondrial Proteome: Multidimensional Separation Techniques for Mitochondrial Proteomics. J. Proteome Res..

[33] Barrientos A. (2003). Yeast Models of Human Mitochondrial Diseases. IUBMB Life.

[34] Rinaldi T., Dallabona C., Ferrero I., Frontali L., Bolotin-Fukuhara M. (2010). Mitochondrial Diseases and the Role of the Yeast Models. FEMS Yeast Res..

[35] Baile M.G., Claypool S.M. (2013). The Power of Yeast to Model Diseases of the Powerhouse of the Cell. Front. Biosci. (Landmark Ed.).

[36] Francisci S., Montanari A. (2017). Mitochondrial Diseases: Yeast as a Model for the Study of Suppressors. Biochim. Biophys. Acta Mol. Cell Res..

[37] Khurana V., Lindquist S. (2010). Modelling Neurodegeneration in *Saccharomyces Cerevisiae*: Why Cook with Baker’s Yeast?. Nat. Rev. Neurosci..

[38] Bourgeron T., Rustin P., Chretien D., Birch-Machin M., Bourgeois M., Viegas-Péquignot E., Munnich A., Rötig A. (1995). Mutation of a Nuclear Succinate Dehydrogenase Gene Results in Mitochondrial Respiratory Chain Deficiency. Nat. Genet..

[39] Alston C.L., Davison J.E., Meloni F., van der Westhuizen F.H., He L., Hornig-Do H.-T., Peet A.C., Gissen P., Goffrini P., Ferrero I. (2012). Recessive Germline SDHA and SDHB Mutations Causing Leukodystrophy and Isolated Mitochondrial Complex II Deficiency. J. Med. Genet..

[40] Nesti C., Meschini M.C., Meunier B., Sacchini M., Doccini S., Romano A., Petrillo S., Pezzini I., Seddiki N., Rubegni A. (2015). Additive Effect of Nuclear and Mitochondrial Mutations in a Patient with Mitochondrial Encephalomyopathy. Hum. Mol. Genet..

[41] Alston C.L., Ceccatelli Berti C., Blakely E.L., Oláhová M., He L., McMahon C.J., Olpin S.E., Hargreaves I.P., Nolli C., McFarland R. (2015). A Recessive Homozygous p.Asp92Gly SDHD Mutation Causes Prenatal Cardiomyopathy and a Severe Mitochondrial Complex II Deficiency. Hum. Genet..

[42] Chang Y.-L., Hsieh M.-H., Chang W.-W., Wang H.-Y., Lin M.-C., Wang C.-P., Lou P.-J., Teng S.-C. (2015). Instability of Succinate Dehydrogenase in SDHD Polymorphism Connects Reactive Oxygen Species Production to Nuclear and Mitochondrial Genomic Mutations in Yeast. Antioxid. Redox Signal..

[43] Massa V., Fernandez-Vizarra E., Alshahwan S., Bakhsh E., Goffrini P., Ferrero I., Mereghetti P., D’Adamo P., Gasparini P., Zeviani M. (2008). Severe Infantile Encephalomyopathy Caused by a Mutation in COX6B1, a Nucleus-Encoded Subunit of Cytochrome c Oxidase. Am. J. Hum. Genet..

[44] Sardin E., Donadello S., di Rago J.P., Tetaud E. (2015). Biochemical investigation of a human pathogenic mutation in the nuclear ATP5E gene using yeast as a model. Front. Genet..

[45] Ghezzi D., Goffrini P., Uziel G., Horvath R., Klopstock T., Lochmüller H., D’Adamo P., Gasparini P., Strom T.M., Prokisch H. (2009). SDHAF1, Encoding a LYR Complex-II Specific Assembly Factor, Is Mutated in SDH-Defective Infantile Leukoencephalopathy. Nat. Genet..

[46] Fernandez-Vizarra E., Bugiani M., Goffrini P., Carrara F., Farina L., Procopio E., Donati A., Uziel G., Ferrero I., Zeviani M. (2007). Impaired Complex III Assembly Associated with BCS1L Gene Mutations in Isolated Mitochondrial Encephalopathy. Hum. Mol. Genet..

[47] Tuppen H.A.L., Fehmi J., Czermin B., Goffrini P., Meloni F., Ferrero I., He L., Blakely E.L., McFarland R., Horvath R. (2010). Long-Term Survival of Neonatal Mitochondrial Complex III Deficiency Associated with a Novel BCS1L Gene Mutation. Mol. Genet. Metab..

[48] de Lonlay P., Valnot I., Barrientos A., Gorbatyuk M., Tzagoloff A., Taanman J.W., Benayoun E., Chrétien D., Kadhom N., Lombès A. (2001). A Mutant Mitochondrial Respiratory Chain Assembly Protein Causes Complex III Deficiency in Patients with Tubulopathy, Encephalopathy and Liver Failure. Nat. Genet..

[49] Meunier B., Fisher N., Ransac S., Mazat J.-P., Brasseur G. (2013). Respiratory Complex III Dysfunction in Humans and the Use of Yeast as a Model Organism to Study Mitochondrial Myopathy and Associated Diseases. Biochim. Biophys. Acta.

[50] Oláhová M., Ceccatelli Berti C., Collier J.J., Alston C.L., Jameson E., Jones S.A., Edwards N., He L., Chinnery P.F., Horvath R. (2019). Molecular Genetic Investigations Identify New Clinical Phenotypes Associated with BCS1L-Related Mitochondrial Disease. Hum. Mol. Genet..

[51] Invernizzi F., Tigano M., Dallabona C., Donnini C., Ferrero I., Cremonte M., Ghezzi D., Lamperti C., Zeviani M. (2013). A Homozygous Mutation in LYRM7/MZM1L Associated with Early Onset Encephalopathy, Lactic Acidosis, and Severe Reduction of Mitochondrial Complex III Activity. Hum. Mutat..

[52] Dallabona C., Abbink T.E.M., Carrozzo R., Torraco A., Legati A., van Berkel C.G.M., Niceta M., Langella T., Verrigni D., Rizza T. (2016). LYRM7 Mutations Cause a Multifocal Cavitating Leukoencephalopathy with Distinct MRI Appearance. Brain.

[53] Valnot I., von Kleist-Retzow J.C., Barrientos A., Gorbatyuk M., Taanman J.W., Mehaye B., Rustin P., Tzagoloff A., Munnich A., Rötig A. (2000). A Mutation in the Human Heme A:Farnesyltransferase Gene (COX10 ) Causes Cytochrome c Oxidase Deficiency. Hum. Mol. Genet..

[54] Pitceathly R.D.S., Taanman J.-W., Rahman S., Meunier B., Sadowski M., Cirak S., Hargreaves I., Land J.M., Nanji T., Polke J.M. (2013). COX10 Mutations Resulting in Complex Multisystem Mitochondrial Disease That Remains Stable into Adulthood. JAMA Neurol..

[55] Reinhold R., Bareth B., Balleininger M., Wissel M., Rehling P., Mick D.U. (2011). Mimicking a SURF1 Allele Reveals Uncoupling of Cytochrome c Oxidase Assembly from Translational Regulation in Yeast. Hum. Mol. Genet..

[56] Bestwick M., Jeong M.-Y., Khalimonchuk O., Kim H., Winge D.R. (2010). Analysis of Leigh Syndrome Mutations in the Yeast SURF1 Homolog Reveals a New Member of the Cytochrome Oxidase Assembly Factor Family. Mol. Cell. Biol..

[57] De Meirleir L., Seneca S., Lissens W., De Clercq I., Eyskens F., Gerlo E., Smet J., Van Coster R. (2004). Respiratory Chain Complex V Deficiency Due to a Mutation in the Assembly Gene ATP12. J. Med. Genet..

[58] Meulemans A., Seneca S., Pribyl T., Smet J., Alderweirldt V., Waeytens A., Lissens W., Van Coster R., De Meirleir L., di Rago J.-P. (2010). Defining the Pathogenesis of the Human Atp12p W94R Mutation Using a *Saccharomyces cerevisiae* Yeast Model. J. Biol. Chem..

[59] Di Bella D., Lazzaro F., Brusco A., Plumari M., Battaglia G., Pastore A., Finardi A., Cagnoli C., Tempia F., Frontali M. (2010). Mutations in the Mitochondrial Protease Gene AFG3L2 Cause Dominant Hereditary Ataxia SCA28. Nat. Genet..

[60] Magri S., Fracasso V., Plumari M., Alfei E., Ghezzi D., Gellera C., Rusmini P., Poletti A., Di Bella D., Elia A.E. (2018). Concurrent AFG3L2 and SPG7 Mutations Associated with Syndromic Parkinsonism and Optic Atrophy with Aberrant OPA1 Processing and Mitochondrial Network Fragmentation. Hum. Mutat..

[61] Pierson T.M., Adams D., Bonn F., Martinelli P., Cherukuri P.F., Teer J.K., Hansen N.F., Cruz P., Mullikin For The Nisc Comparative Sequencing Program J.C., Blakesley R.W. (2011). Whole-Exome Sequencing Identifies Homozygous AFG3L2 Mutations in a Spastic Ataxia-Neuropathy Syndrome Linked to Mitochondrial m-AAA Proteases. PLoS Genet..

[62] Bonn F., Pantakani K., Shoukier M., Langer T., Mannan A.U. (2010). Functional Evaluation of Paraplegin Mutations by a Yeast Complementation Assay. Hum. Mutat..

[63] Atorino L., Silvestri L., Koppen M., Cassina L., Ballabio A., Marconi R., Langer T., Casari G. (2003). Loss of M-AAA Protease in Mitochondria Causes Complex I Deficiency and Increased Sensitivity to Oxidative Stress in Hereditary Spastic Paraplegia. J. Cell Biol..

[64] Koppen M., Metodiev M.D., Casari G., Rugarli E.I., Langer T. (2007). Variable and Tissue-Specific Subunit Composition of Mitochondrial m-AAA Protease Complexes Linked to Hereditary Spastic Paraplegia. Mol. Cell. Biol..

[65] Di Fonzo A., Ronchi D., Lodi T., Fassone E., Tigano M., Lamperti C., Corti S., Bordoni A., Fortunato F., Nizzardo M. (2009). The Mitochondrial Disulfide Relay System Protein GFER Is Mutated in Autosomal-Recessive Myopathy with Cataract and Combined Respiratory-Chain Deficiency. Am. J. Hum. Genet..

[66] Ceh-Pavia E., Ang S.K., Spiller M.P., Lu H. (2014). The Disease-Associated Mutation of the Mitochondrial Thiol Oxidase Erv1 Impairs Cofactor Binding during Its Catalytic Reaction. Biochem. J..

[67] Eldomery M.K., Akdemir Z.C., Vögtle F.-N., Charng W.-L., Mulica P., Rosenfeld J.A., Gambin T., Gu S., Burrage L.C., Al Shamsi A. (2016). MIPEP Recessive Variants Cause a Syndrome of Left Ventricular Non-Compaction, Hypotonia, and Infantile Death. Genome Med..

[68] Brunetti D., Torsvik J., Dallabona C., Teixeira P., Sztromwasser P., Fernandez-Vizarra E., Cerutti R., Reyes A., Preziuso C., D’Amati G. (2016). Defective PITRM1 Mitochondrial Peptidase Is Associated with Aβ Amyloidotic Neurodegeneration. EMBO Mol. Med..

[69] Langer Y., Aran A., Gulsuner S., Abu Libdeh B., Renbaum P., Brunetti D., Teixeira P.-F., Walsh T., Zeligson S., Ruotolo R. (2018). Mitochondrial PITRM1 Peptidase Loss-of-Function in Childhood Cerebellar Atrophy. J. Med. Genet..

[70] Vögtle F.-N., Brändl B., Larson A., Pendziwiat M., Friederich M.W., White S.M., Basinger A., Kücükköse C., Muhle H., Jähn J.A. (2018). Mutations in PMPCB Encoding the Catalytic Subunit of the Mitochondrial Presequence Protease Cause Neurodegeneration in Early Childhood. Am. J. Hum. Genet..

[71] Shahrour M.A., Staretz-Chacham O., Dayan D., Stephen J., Weech A., Damseh N., Pri Chen H., Edvardson S., Mazaheri S., Saada A. (2017). Mitochondrial Epileptic Encephalopathy, 3-Methylglutaconic Aciduria and Variable Complex V Deficiency Associated with TIMM50 Mutations. Clin. Genet..

[72] Roesch K., Curran S.P., Tranebjaerg L., Koehler C.M. (2002). Human Deafness Dystonia Syndrome Is Caused by a Defect in Assembly of the DDP1/TIMM8a-TIMM13 Complex. Hum. Mol. Genet..

[73] Hofmann S., Rothbauer U., Mühlenbein N., Neupert W., Gerbitz K.-D., Brunner M., Bauer M.F. (2002). The C66W Mutation in the Deafness Dystonia Peptide 1 (DDP1) Affects the Formation of Functional DDP1.TIM13 Complexes in the Mitochondrial Intermembrane Space. J. Biol. Chem..

[74] Spinazzola A., Viscomi C., Fernandez-Vizarra E., Carrara F., D’Adamo P., Calvo S., Marsano R.M., Donnini C., Weiher H., Strisciuglio P. (2006). MPV17 Encodes an Inner Mitochondrial Membrane Protein and Is Mutated in Infantile Hepatic Mitochondrial DNA Depletion. Nat. Genet..

[75] Gilberti M., Baruffini E., Donnini C., Dallabona C. (2018). Pathological Alleles of MPV17 Modeled in the Yeast *Saccharomyces cerevisiae* Orthologous Gene SYM1 Reveal Their Inability to Take Part in a High Molecular Weight Complex. PLoS ONE.

[76] Stuart G.R., Santos J.H., Strand M.K., Van Houten B., Copeland W.C. (2006). Mitochondrial and Nuclear DNA Defects in *Saccharomyces cerevisiae* with Mutations in DNA Polymerase Gamma Associated with Progressive External Ophthalmoplegia. Hum. Mol. Genet..

[77] Baruffini E., Lodi T., Dallabona C., Puglisi A., Zeviani M., Ferrero I. (2006). Genetic and Chemical Rescue of the *Saccharomyces cerevisiae* Phenotype Induced by Mitochondrial DNA Polymerase Mutations Associated with Progressive External Ophthalmoplegia in Humans. Hum. Mol. Genet..

[78] Baruffini E., Ferrero I., Foury F. (2007). Mitochondrial DNA Defects in *Saccharomyces cerevisiae* Caused by Functional Interactions between DNA Polymerase Gamma Mutations Associated with Disease in Human. Biochim. Biophys. Acta.

[79] Szczepanowska K., Foury F. (2010). A Cluster of Pathogenic Mutations in the 3’-5’ Exonuclease Domain of DNA Polymerase Gamma Defines a Novel Module Coupling DNA Synthesis and Degradation. Hum. Mol. Genet..

[80] Stricker S., Prüss H., Horvath R., Baruffini E., Lodi T., Siebert E., Endres M., Zschenderlein R., Meisel A. (2009). A Variable Neurodegenerative Phenotype with Polymerase Gamma Mutation. J. Neurol. Neurosurg. Psychiatry.

[81] Stumpf J.D., Bailey C.M., Spell D., Stillwagon M., Anderson K.S., Copeland W.C. (2010). Mip1 Containing Mutations Associated with Mitochondrial Disease Causes Mutagenesis and Depletion of MtDNA in *Saccharomyces cerevisiae*. Hum. Mol. Genet..

[82] Stewart J.D., Horvath R., Baruffini E., Ferrero I., Bulst S., Watkins P.B., Fontana R.J., Day C.P., Chinnery P.F. (2010). Polymerase γ Gene POLG Determines the Risk of Sodium Valproate-Induced Liver Toxicity. Hepatology.

[83] Baruffini E., Horvath R., Dallabona C., Czermin B., Lamantea E., Bindoff L., Invernizzi F., Ferrero I., Zeviani M., Lodi T. (2011). Predicting the Contribution of Novel POLG Mutations to Human Disease through Analysis in Yeast Model. Mitochondrion.

[84] Baruffini E., Serafini F., Ferrero I., Lodi T. (2012). Overexpression of DNA Polymerase Zeta Reduces the Mitochondrial Mutability Caused by Pathological Mutations in DNA Polymerase Gamma in Yeast. PLoS ONE.

[85] Stumpf J.D., Copeland W.C. (2013). The Exonuclease Activity of the Yeast Mitochondrial DNA Polymerase γ Suppresses Mitochondrial DNA Deletions between Short Direct Repeats in *Saccharomyces cerevisiae*. Genetics.

[86] Stumpf J.D., Copeland W.C. (2014). MMS Exposure Promotes Increased MtDNA Mutagenesis in the Presence of Replication-Defective Disease-Associated DNA Polymerase γ Variants. PLoS Genet..

[87] Kaliszewska M., Kruszewski J., Kierdaszuk B., Kostera-Pruszczyk A., Nojszewska M., Łusakowska A., Vizueta J., Sabat D., Lutyk D., Lower M. (2015). Yeast Model Analysis of Novel Polymerase Gamma Variants Found in Patients with Autosomal Recessive Mitochondrial Disease. Hum. Genet..

[88] Hoyos-Gonzalez N., Trasviña-Arenas C.H., Degiorgi A., Castro-Lara A.Y., Peralta-Castro A., Jimenez-Sandoval P., Diaz-Quezada C., Lodi T., Baruffini E., Brieba L.G. (2020). Modeling of Pathogenic Variants of Mitochondrial DNA Polymerase: Insight into the Replication Defects and Implication for Human Disease. Biochim. Biophys. Acta Gen. Subj..

[89] Qian Y., Kachroo A.H., Yellman C.M., Marcotte E.M., Johnson K.A. (2014). Yeast Cells Expressing the Human Mitochondrial DNA Polymerase Reveal Correlations between Polymerase Fidelity and Human Disease Progression. J. Biol. Chem..

[90] Qian Y., Ziehr J.L., Johnson K.A. (2015). Alpers Disease Mutations in Human DNA Polymerase Gamma Cause Catalytic Defects in Mitochondrial DNA Replication by Distinct Mechanisms. Front. Genet..

[91] Haijes H.A., Koster M.J.E., Rehmann H., Li D., Hakonarson H., Cappuccio G., Hancarova M., Lehalle D., Reardon W., Schaefer G.B. (2019). De Novo Heterozygous POLR2A Variants Cause a Neurodevelopmental Syndrome with Profound Infantile-Onset Hypotonia. Am. J. Hum. Genet..

[92] Haack T.B., Kopajtich R., Freisinger P., Wieland T., Rorbach J., Nicholls T.J., Baruffini E., Walther A., Danhauser K., Zimmermann F.A. (2013). ELAC2 Mutations Cause a Mitochondrial RNA Processing Defect Associated with Hypertrophic Cardiomyopathy. Am. J. Hum. Genet..

[93] Li X., Guan M.-X. (2002). A Human Mitochondrial GTP Binding Protein Related to TRNA Modification May Modulate Phenotypic Expression of the Deafness-Associated Mitochondrial 12S RRNA Mutation. Mol. Cell. Biol..

[94] Garone C., D’Souza A.R., Dallabona C., Lodi T., Rebelo-Guiomar P., Rorbach J., Donati M.A., Procopio E., Montomoli M., Guerrini R. (2017). Defective Mitochondrial RRNA Methyltransferase MRM2 Causes MELAS-like Clinical Syndrome. Hum. Mol. Genet..

[95] Baruffini E., Dallabona C., Invernizzi F., Yarham J.W., Melchionda L., Blakely E.L., Lamantea E., Donnini C., Santra S., Vijayaraghavan S. (2013). MTO1 Mutations Are Associated with Hypertrophic Cardiomyopathy and Lactic Acidosis and Cause Respiratory Chain Deficiency in Humans and Yeast. Hum. Mutat..

[96] Ghezzi D., Baruffini E., Haack T.B., Invernizzi F., Melchionda L., Dallabona C., Strom T.M., Parini R., Burlina A.B., Meitinger T. (2012). Mutations of the Mitochondrial-TRNA Modifier MTO1 Cause Hypertrophic Cardiomyopathy and Lactic Acidosis. Am. J. Hum. Genet..

[97] Yarham J.W., Lamichhane T.N., Pyle A., Mattijssen S., Baruffini E., Bruni F., Donnini C., Vassilev A., He L., Blakely E.L. (2014). Defective I6A37 Modification of Mitochondrial and Cytosolic TRNAs Results from Pathogenic Mutations in TRIT1 and Its Substrate TRNA. PLoS Genet..

[98] Powell C.A., Kopajtich R., D’Souza A.R., Rorbach J., Kremer L.S., Husain R.A., Dallabona C., Donnini C., Alston C.L., Griffin H. (2015). TRMT5 Mutations Cause a Defect in Post-Transcriptional Modification of Mitochondrial TRNA Associated with Multiple Respiratory-Chain Deficiencies. Am. J. Hum. Genet..

[99] Yan Q., Li X., Faye G., Guan M.-X. (2005). Mutations in MTO2 Related to TRNA Modification Impair Mitochondrial Gene Expression and Protein Synthesis in the Presence of a Paromomycin Resistance Mutation in Mitochondrial 15 S RRNA. J. Biol. Chem..

[100] Umeda N., Suzuki T., Yukawa M., Ohya Y., Shindo H., Watanabe K., Suzuki T. (2005). Mitochondria-Specific RNA-Modifying Enzymes Responsible for the Biosynthesis of the Wobble Base in Mitochondrial TRNAs. Implications for the Molecular Pathogenesis of Human Mitochondrial Diseases. J. Biol. Chem..

[101] Chakraborty P.K., Schmitz-Abe K., Kennedy E.K., Mamady H., Naas T., Durie D., Campagna D.R., Lau A., Sendamarai A.K., Wiseman D.H. (2014). Mutations in TRNT1 Cause Congenital Sideroblastic Anemia with Immunodeficiency, Fevers, and Developmental Delay (SIFD). Blood.

[102] Leibovitch M., Reid N.E., Victoria J., Hanic-Joyce P.J., Joyce P.B.M. (2019). Analysis of the Pathogenic I326T Variant of Human TRNA Nucleotidyltransferase Reveals Reduced Catalytic Activity and Thermal Stability in Vitro Linked to a Conformational Change. Biochim. Biophys. Acta Proteins Proteom..

[103] Dallabona C., Diodato D., Kevelam S.H., Haack T.B., Wong L.-J., Salomons G.S., Baruffini E., Melchionda L., Mariotti C., Strom T.M. (2014). Novel (Ovario) Leukodystrophy Related to AARS2 Mutations. Neurology.

[104] Kuo M.E., Antonellis A., Shakkottai V.G. (2020). Alanyl-TRNA Synthetase 2 (AARS2)-Related Ataxia Without Leukoencephalopathy. Cerebellum.

[105] Griffin L.B., Sakaguchi R., McGuigan D., Gonzalez M.A., Searby C., Züchner S., Hou Y.-M., Antonellis A. (2014). Impaired Function Is a Common Feature of Neuropathy-Associated Glycyl-TRNA Synthetase Mutations. Hum. Mutat.

[106] Lee D.C., Meyer-Schuman R., Bacon C., Shy M.E., Antonellis A., Scherer S.S. (2019). A Recurrent GARS Mutation Causes Distal Hereditary Motor Neuropathy. J. Peripher. Nerv. Syst..

[107] Markovitz R., Ghosh R., Kuo M.E., Hong W., Lim J., Bernes S., Manberg S., Crosby K., Tanpaiboon P., Bharucha-Goebel D. (2020). GARS-Related Disease in Infantile Spinal Muscular Atrophy: Implications for Diagnosis and Treatment. Am. J. Med. Genet. A.

[108] Friederich M.W., Timal S., Powell C.A., Dallabona C., Kurolap A., Palacios-Zambrano S., Bratkovic D., Derks T.G.J., Bick D., Bouman K. (2018). Pathogenic Variants in Glutamyl-TRNAGln Amidotransferase Subunits Cause a Lethal Mitochondrial Cardiomyopathy Disorder. Nat. Commun..

[109] Pierce S.B., Chisholm K.M., Lynch E.D., Lee M.K., Walsh T., Opitz J.M., Li W., Klevit R.E., King M.-C. (2011). Mutations in Mitochondrial Histidyl TRNA Synthetase HARS2 Cause Ovarian Dysgenesis and Sensorineural Hearing Loss of Perrault Syndrome. Proc. Natl. Acad. Sci. USA.

[110] Wang Y., Zhou J.-B., Zeng Q.-Y., Wu S., Xue M.-Q., Fang P., Wang E.-D., Zhou X.-L. (2020). Hearing Impairment-Associated KARS Mutations Lead to Defects in Aminoacylation of Both Cytoplasmic and Mitochondrial TRNALys. Sci. China Life Sci..

[111] Pierce S.B., Gersak K., Michaelson-Cohen R., Walsh T., Lee M.K., Malach D., Klevit R.E., King M.-C., Levy-Lahad E. (2013). Mutations in LARS2, Encoding Mitochondrial Leucyl-TRNA Synthetase, Lead to Premature Ovarian Failure and Hearing Loss in Perrault Syndrome. Am. J. Hum. Genet..

[112] Cassandrini D., Cilio M.R., Bianchi M., Doimo M., Balestri M., Tessa A., Rizza T., Sartori G., Meschini M.C., Nesti C. (2013). Pontocerebellar Hypoplasia Type 6 Caused by Mutations in RARS2: Definition of the Clinical Spectrum and Molecular Findings in Five Patients. J. Inherit. Metab. Dis..

[113] Wang Y., Zhou X.-L., Ruan Z.-R., Liu R.-J., Eriani G., Wang E.-D. (2016). A Human Disease-Causing Point Mutation in Mitochondrial Threonyl-TRNA Synthetase Induces Both Structural and Functional Defects. J. Biol. Chem..

[114] Diodato D., Melchionda L., Haack T.B., Dallabona C., Baruffini E., Donnini C., Granata T., Ragona F., Balestri P., Margollicci M. (2014). VARS2 and TARS2 Mutations in Patients with Mitochondrial Encephalomyopathies. Hum. Mutat..

[115] Chin H.-L., Goh D.L.-M., Wang F.S., Tay S.K.H., Heng C.K., Donnini C., Baruffini E., Pines O. (2019). A Combination of Two Novel VARS2 Variants Causes a Mitochondrial Disorder Associated with Failure to Thrive and Pulmonary Hypertension. J. Mol. Med..

[116] Maffezzini C., Laine I., Dallabona C., Clemente P., Calvo-Garrido J., Wibom R., Naess K., Barbaro M., Falk A., Donnini C. (2019). Mutations in the Mitochondrial Tryptophanyl-TRNA Synthetase Cause Growth Retardation and Progressive Leukoencephalopathy. Mol. Genet. Genom. Med..

[117] Ardissone A., Lamantea E., Quartararo J., Dallabona C., Carrara F., Moroni I., Donnini C., Garavaglia B., Zeviani M., Uziel G. (2015). A Novel Homozygous YARS2 Mutation in Two Italian Siblings and a Review of Literature. JIMD Rep..

[118] Sommerville E.W., Ng Y.S., Alston C.L., Dallabona C., Gilberti M., He L., Knowles C., Chin S.L., Schaefer A.M., Falkous G. (2017). Clinical Features, Molecular Heterogeneity, and Prognostic Implications in YARS2-Related Mitochondrial Myopathy. JAMA Neurol..

[119] Smith F., Hopton S., Dallabona C., Gilberti M., Falkous G., Norwood F., Donnini C., Gorman G.S., Clark B., Taylor R.W. (2018). Sideroblastic Anemia with Myopathy Secondary to Novel, Pathogenic Missense Variants in the YARS2 Gene. Haematologica.

[120] Valente L., Tiranti V., Marsano R.M., Malfatti E., Fernandez-Vizarra E., Donnini C., Mereghetti P., De Gioia L., Burlina A., Castellan C. (2007). Infantile Encephalopathy and Defective Mitochondrial DNA Translation in Patients with Mutations of Mitochondrial Elongation Factors EFG1 and EFTu. Am. J. Hum. Genet..

[121] Montanari A., Zhou Y.F., D’Orsi M.F., Bolotin-Fukuhara M., Frontali L., Francisci S. (2013). Analyzing the Suppression of Respiratory Defects in the Yeast Model of Human Mitochondrial TRNA Diseases. Gene.

[122] Di Nottia M., Montanari A., Verrigni D., Oliva R., Torraco A., Fernandez-Vizarra E., Diodato D., Rizza T., Bianchi M., Catteruccia M. (2017). Novel Mutation in Mitochondrial Elongation Factor EF-Tu Associated to Dysplastic Leukoencephalopathy and Defective Mitochondrial DNA Translation. Biochim. Biophys. Acta Mol. Basis Dis..

[123] Nasca A., Legati A., Baruffini E., Nolli C., Moroni I., Ardissone A., Goffrini P., Ghezzi D. (2016). Biallelic Mutations in DNM1L Are Associated with a Slowly Progressive Infantile Encephalopathy. Hum. Mutat..

[124] Verrigni D., Di Nottia M., Ardissone A., Baruffini E., Nasca A., Legati A., Bellacchio E., Fagiolari G., Martinelli D., Fusco L. (2019). Clinical-Genetic Features and Peculiar Muscle Histopathology in Infantile DNM1L-Related Mitochondrial Epileptic Encephalopathy. Hum. Mutat..

[125] Ashrafian H., Docherty L., Leo V., Towlson C., Neilan M., Steeples V., Lygate C.A., Hough T., Townsend S., Williams D. (2010). A Mutation in the Mitochondrial Fission Gene Dnm1l Leads to Cardiomyopathy. PLoS Genet..

[126] Amiott E.A., Cohen M.M., Saint-Georges Y., Weissman A.M., Shaw J.M. (2009). A Mutation Associated with CMT2A Neuropathy Causes Defects in Fzo1 GTP Hydrolysis, Ubiquitylation, and Protein Turnover. Mol. Biol. Cell.

[127] Del Dotto V., Fogazza M., Musiani F., Maresca A., Aleo S.J., Caporali L., La Morgia C., Nolli C., Lodi T., Goffrini P. (2018). Deciphering OPA1 Mutations Pathogenicity by Combined Analysis of Human, Mouse and Yeast Cell Models. Biochim. Biophys. Acta Mol. Basis Dis..

[128] Nolli C., Goffrini P., Lazzaretti M., Zanna C., Vitale R., Lodi T., Baruffini E. (2015). Validation of a MGM1/OPA1 Chimeric Gene for Functional Analysis in Yeast of Mutations Associated with Dominant Optic Atrophy. Mitochondrion.

[129] Gu Z., Valianpour F., Chen S., Vaz F.M., Hakkaart G.A., Wanders R.J.A., Greenberg M.L. (2004). Aberrant Cardiolipin Metabolism in the Yeast Taz1 Mutant: A Model for Barth Syndrome. Mol. Microbiol..

[130] Claypool S.M., Whited K., Srijumnong S., Han X., Koehler C.M. (2011). Barth Syndrome Mutations That Cause Tafazzin Complex Lability. J. Cell Biol..

[131] Whited K., Baile M.G., Currier P., Claypool S.M. (2013). Seven Functional Classes of Barth Syndrome Mutation. Hum. Mol. Genet..

[132] Park J.-S., Thorsness M.K., Policastro R., McGoldrick L.L., Hollingsworth N.M., Thorsness P.E., Neiman A.M. (2016). Yeast Vps13 Promotes Mitochondrial Function and Is Localized at Membrane Contact Sites. Mol. Biol. Cell.

[133] Rzepnikowska W., Flis K., Kaminska J., Grynberg M., Urbanek A., Ayscough K.R., Zoladek T. (2017). Amino Acid Substitution Equivalent to Human Chorea-Acanthocytosis I2771R in Yeast Vps13 Protein Affects Its Binding to Phosphatidylinositol 3-Phosphate. Hum. Mol. Genet..

[134] Soczewka P., Kolakowski D., Smaczynska-de Rooij I., Rzepnikowska W., Ayscough K.R., Kaminska J., Zoladek T. (2019). Yeast-Model-Based Study Identified Myosin- and Calcium-Dependent Calmodulin Signalling as a Potential Target for Drug Intervention in Chorea-Acanthocytosis. Dis. Model. Mech..

[135] Benincá C., Zanette V., Brischigliaro M., Johnson M., Reyes A., do Valle D.A., Robinson A.J., Degiorgi A., Yeates A., Telles B.A. (2020). Mutation in the MICOS Subunit Gene APOO (MIC26) Associated with an X-Linked Recessive Mitochondrial Myopathy, Lactic Acidosis, Cognitive Impairment and Autistic Features. J. Med. Genet..

[136] Rzepnikowska W., Kaminska J., Kabzińska D., Kochański A. (2020). Pathogenic Effect of GDAP1 Gene Mutations in a Yeast Model. Genes.

[137] Spiegel R., Pines O., Ta-Shma A., Burak E., Shaag A., Halvardson J., Edvardson S., Mahajna M., Zenvirt S., Saada A. (2012). Infantile Cerebellar-Retinal Degeneration Associated with a Mutation in Mitochondrial Aconitase, ACO2. Am. J. Hum. Genet..

[138] Metodiev M.D., Gerber S., Hubert L., Delahodde A., Chretien D., Gérard X., Amati-Bonneau P., Giacomotto M.-C., Boddaert N., Kaminska A. (2014). Mutations in the Tricarboxylic Acid Cycle Enzyme, Aconitase 2, Cause Either Isolated or Syndromic Optic Neuropathy with Encephalopathy and Cerebellar Atrophy. J. Med. Genet..

[139] Neumann M.A.-C., Grossmann D., Schimpf-Linzenbold S., Dayan D., Stingl K., Ben-Menachem R., Pines O., Massart F., Delcambre S., Ghelfi J. (2020). Haploinsufficiency Due to a Novel ACO2 Deletion Causes Mitochondrial Dysfunction in Fibroblasts from a Patient with Dominant Optic Nerve Atrophy. Sci. Rep..

[140] Sharkia R., Wierenga K.J., Kessel A., Azem A., Bertini E., Carrozzo R., Torraco A., Goffrini P., Ceccatelli Berti C., McCormick M.E. (2019). Clinical, Radiological, and Genetic Characteristics of 16 Patients with ACO2 Gene Defects: Delineation of an Emerging Neurometabolic Syndrome. J. Inherit. Metab. Dis..

[141] Fattal-Valevski A., Eliyahu H., Fraenkel N.D., Elmaliach G., Hausman-Kedem M., Shaag A., Mandel D., Pines O., Elpeleg O. (2017). Homozygous Mutation, p.Pro304His, in IDH3A, Encoding Isocitrate Dehydrogenase Subunit Is Associated with Severe Encephalopathy in Infancy. Neurogenetics.

[142] Ait-El-Mkadem S., Dayem-Quere M., Gusic M., Chaussenot A., Bannwarth S., François B., Genin E.C., Fragaki K., Volker-Touw C.L.M., Vasnier C. (2017). Mutations in MDH2, Encoding a Krebs Cycle Enzyme, Cause Early-Onset Severe Encephalopathy. Am. J. Hum. Genet..

[143] Heimer G., Kerätär J.M., Riley L.G., Balasubramaniam S., Eyal E., Pietikäinen L.P., Hiltunen J.K., Marek-Yagel D., Hamada J., Gregory A. (2016). MECR Mutations Cause Childhood-Onset Dystonia and Optic Atrophy, a Mitochondrial Fatty Acid Synthesis Disorder. Am. J. Hum. Genet..

[144] Bricker D.K., Taylor E.B., Schell J.C., Orsak T., Boutron A., Chen Y.-C., Cox J.E., Cardon C.M., Van Vranken J.G., Dephoure N. (2012). A Mitochondrial Pyruvate Carrier Required for Pyruvate Uptake in Yeast, Drosophila, and Humans. Science.

[145] Drakulic S., Rai J., Petersen S.V., Golas M.M., Sander B. (2018). Folding and Assembly Defects of Pyruvate Dehydrogenase Deficiency-Related Variants in the E1α Subunit of the Pyruvate Dehydrogenase Complex. Cell. Mol. Life Sci..

[146] Aral B., Benelli C., Ait-Ghezala G., Amessou M., Fouque F., Maunoury C., Créau N., Kamoun P., Marsac C. (1997). Mutations in PDX1, the Human Lipoyl-Containing Component X of the Pyruvate Dehydrogenase-Complex Gene on Chromosome 11p1, in Congenital Lactic Acidosis. Am. J. Hum. Genet..

[147] Guimier A., Gordon C.T., Godard F., Ravenscroft G., Oufadem M., Vasnier C., Rambaud C., Nitschke P., Bole-Feysot C., Masson C. (2016). Biallelic PPA2 Mutations Cause Sudden Unexpected Cardiac Arrest in Infancy. Am. J. Hum. Genet..

[148] Zeng H.-S., Zhao S.-T., Deng M., Zhang Z.-H., Cai X.-R., Chen F.-P., Song Y.-Z. (2014). Inspissated Bile Syndrome in an Infant with Citrin Deficiency and Congenital Anomalies of the Biliary Tract and Esophagus: Identification and Pathogenicity Analysis of a Novel SLC25A13 Mutation with Incomplete Penetrance. Int. J. Mol. Med..

[149] Seifert E.L., Gál A., Acoba M.G., Li Q., Anderson-Pullinger L., Golenár T., Moffat C., Sondheimer N., Claypool S.M., Hajnóczky G. (2016). Natural and Induced Mitochondrial Phosphate Carrier Loss: Differential dependence of mitochondrial metabolism and dynamics and cell survival on the extent of depletion. J. Biol. Chem..

[150] Mayr J.A., Merkel O., Kohlwein S.D., Gebhardt B.R., Böhles H., Fötschl U., Koch J., Jaksch M., Lochmüller H., Horváth R. (2007). Mitochondrial Phosphate-Carrier Deficiency: A Novel Disorder of Oxidative Phosphorylation. Am. J. Hum. Genet..

[151] Allikmets R., Raskind W.H., Hutchinson A., Schueck N.D., Dean M., Koeller D.M. (1999). Mutation of a Putative Mitochondrial Iron Transporter Gene (ABC7) in X-Linked Sideroblastic Anemia and Ataxia (XLSA/A). Hum. Mol. Genet..

[152] Pearson S.A., Wachnowsky C., Cowan J.A. (2020). Defining the Mechanism of the Mitochondrial Atm1p [2Fe-2S] Cluster Exporter. Metallomics.

[153] Bekri S., Kispal G., Lange H., Fitzsimons E., Tolmie J., Lill R., Bishop D.F. (2000). Human ABC7 Transporter: Gene Structure and Mutation Causing X-Linked Sideroblastic Anemia with Ataxia with Disruption of Cytosolic Iron-Sulfur Protein Maturation. Blood.

[154] Paul A., Drecourt A., Petit F., Deguine D.D., Vasnier C., Oufadem M., Masson C., Bonnet C., Masmoudi S., Mosnier I. (2017). FDXR Mutations Cause Sensorial Neuropathies and Expand the Spectrum of Mitochondrial Fe-S-Synthesis Diseases. Am. J. Hum. Genet..

[155] Cavadini P., Gellera C., Patel P.I., Isaya G. (2000). Human Frataxin Maintains Mitochondrial Iron Homeostasis in *Saccharomyces cerevisiae*. Hum. Mol. Genet..

[156] Wilson R.B., Roof D.M. (1997). Respiratory Deficiency Due to Loss of Mitochondrial DNA in Yeast Lacking the Frataxin Homologue. Nat. Genet..

[157] Foury F. (2007). Screens for Mitochondrial Mutants in Yeast. Methods Mol. Biol..

[158] Kim H.-M., Narayanan V., Mieczkowski P.A., Petes T.D., Krasilnikova M.M., Mirkin S.M., Lobachev K.S. (2008). Chromosome Fragility at GAA Tracts in Yeast Depends on Repeat Orientation and Requires Mismatch Repair. EMBO J..

[159] Leidgens S., De Smet S., Foury F. (2010). Frataxin Interacts with Isu1 through a Conserved Tryptophan in Its Beta-Sheet. Hum. Mol. Genet..

[160] Khristich A.N., Armenia J.F., Matera R.M., Kolchinski A.A., Mirkin S.M. (2020). Large-Scale Contractions of Friedreich’s Ataxia GAA Repeats in Yeast Occur during DNA Replication Due to Their Triplex-Forming Ability. Proc. Natl. Acad. Sci. USA.

[161] Saha P.P., Kumar S.K.P., Srivastava S., Sinha D., Pareek G., D’Silva P. (2014). The Presence of Multiple Cellular Defects Associated with a Novel G50E Iron-Sulfur Cluster Scaffold Protein (ISCU) Mutation Leads to Development of Mitochondrial Myopathy. J. Biol. Chem..

[162] Legati A., Reyes A., Ceccatelli Berti C., Stehling O., Marchet S., Lamperti C., Ferrari A., Robinson A.J., Mühlenhoff U., Lill R. (2017). A Novel de Novo Dominant Mutation in ISCU Associated with Mitochondrial Myopathy. J. Med. Genet..

[163] Lim S.C., Friemel M., Marum J.E., Tucker E.J., Bruno D.L., Riley L.G., Christodoulou J., Kirk E.P., Boneh A., DeGennaro C.M. (2013). Mutations in LYRM4, Encoding Iron-Sulfur Cluster Biogenesis Factor ISD11, Cause Deficiency of Multiple Respiratory Chain Complexes. Hum. Mol. Genet..

[164] Navarro-Sastre A., Tort F., Stehling O., Uzarska M.A., Arranz J.A., Del Toro M., Labayru M.T., Landa J., Font A., Garcia-Villoria J. (2011). A Fatal Mitochondrial Disease Is Associated with Defective NFU1 Function in the Maturation of a Subset of Mitochondrial Fe-S Proteins. Am. J. Hum. Genet..

[165] Dusi S., Valletta L., Haack T.B., Tsuchiya Y., Venco P., Pasqualato S., Goffrini P., Tigano M., Demchenko N., Wieland T. (2014). Exome Sequence Reveals Mutations in CoA Synthase as a Cause of Neurodegeneration with Brain Iron Accumulation. Am. J. Hum. Genet..

[166] Ceccatelli Berti C., Dallabona C., Lazzaretti M., Dusi S., Tosi E., Tiranti V., Goffrini P. (2015). Modeling Human Coenzyme A Synthase Mutation in Yeast Reveals Altered Mitochondrial Function, Lipid Content and Iron Metabolism. Microb. Cell.

[167] Soreze Y., Boutron A., Habarou F., Barnerias C., Nonnenmacher L., Delpech H., Mamoune A., Chrétien D., Hubert L., Bole-Feysot C. (2013). Mutations in Human Lipoyltransferase Gene LIPT1 Cause a Leigh Disease with Secondary Deficiency for Pyruvate and Alpha-Ketoglutarate Dehydrogenase. Orphanet J. Rare Dis..

[168] Habarou F., Hamel Y., Haack T.B., Feichtinger R.G., Lebigot E., Marquardt I., Busiah K., Laroche C., Madrange M., Grisel C. (2017). Biallelic Mutations in LIPT2 Cause a Mitochondrial Lipoylation Defect Associated with Severe Neonatal Encephalopathy. Am. J. Hum. Genet..

[169] Ceccatelli Berti C., Gilea A.I., De Gregorio M.A., Goffrini P. (2020). Exploring Yeast as a Study Model of Pantothenate Kinase-Associated Neurodegeneration and for the Identification of Therapeutic Compounds. Int. J. Mol. Sci.

[170] Edvardson S., Porcelli V., Jalas C., Soiferman D., Kellner Y., Shaag A., Korman S.H., Pierri C.L., Scarcia P., Fraenkel N.D. (2013). Agenesis of Corpus Callosum and Optic Nerve Hypoplasia Due to Mutations in SLC25A1 Encoding the Mitochondrial Citrate Transporter. J. Med. Genet..

[171] Palmieri L., Alberio S., Pisano I., Lodi T., Meznaric-Petrusa M., Zidar J., Santoro A., Scarcia P., Fontanesi F., Lamantea E. (2005). Complete Loss-of-Function of the Heart/Muscle-Specific Adenine Nucleotide Translocator Is Associated with Mitochondrial Myopathy and Cardiomyopathy. Hum. Mol. Genet..

[172] Chen X.J. (2002). Induction of an Unregulated Channel by Mutations in Adenine Nucleotide Translocase Suggests an Explanation for Human Ophthalmoplegia. Hum. Mol. Genet..

[173] Fontanesi F., Palmieri L., Scarcia P., Lodi T., Donnini C., Limongelli A., Tiranti V., Zeviani M., Ferrero I., Viola A.M. (2004). Mutations in AAC2, Equivalent to Human AdPEO-Associated ANT1 Mutations, Lead to Defective Oxidative Phosphorylation in *Saccharomyces cerevisiae* and Affect Mitochondrial DNA Stability. Hum. Mol. Genet..

[174] Lodi T., Bove C., Fontanesi F., Viola A.M., Ferrero I. (2006). Mutation D104G in ANT1 Gene: Complementation Study in *Saccharomyces cerevisiae* as a Model System. Biochem. Biophys Res. Commun..

[175] Liu Y., Wang X., Chen X.J. (2015). Misfolding of Mutant Adenine Nucleotide Translocase in Yeast Supports a Novel Mechanism of Ant1-Induced Muscle Diseases. Mol. Biol. Cell.

[176] Wang X., Salinas K., Zuo X., Kucejova B., Chen X.J. (2008). Dominant Membrane Uncoupling by Mutant Adenine Nucleotide Translocase in Mitochondrial Diseases. Hum. Mol. Genet..

[177] Kaukonen J., Juselius J.K., Tiranti V., Kyttälä A., Zeviani M., Comi G.P., Keränen S., Peltonen L., Suomalainen A. (2000). Role of Adenine Nucleotide Translocator 1 in MtDNA Maintenance. Science.

[178] Dallabona C., Baruffini E., Goffrini P., Lodi T. (2017). Dominance of Yeast Aac2R96H and Aac2R252G Mutations, Equivalent to Pathological Mutations in Ant1, Is Due to Gain of Function. Biochem. Biophys Res. Commun..

[179] Thompson K., Majd H., Dallabona C., Reinson K., King M.S., Alston C.L., He L., Lodi T., Jones S.A., Fattal-Valevski A. (2016). Recurrent De Novo Dominant Mutations in SLC25A4 Cause Severe Early-Onset Mitochondrial Disease and Loss of Mitochondrial DNA Copy Number. Am. J. Hum. Genet..

[180] Spiegel R., Shaag A., Edvardson S., Mandel H., Stepensky P., Shalev S.A., Horovitz Y., Pines O., Elpeleg O. (2009). SLC25A19 Mutation as a Cause of Neuropathy and Bilateral Striatal Necrosis. Ann. Neurol..

[181] Schiff M., Veauville-Merllié A., Su C.H., Tzagoloff A., Rak M., Ogier de Baulny H., Boutron A., Smedts-Walters H., Romero N.B., Rigal O. (2016). SLC25A32 Mutations and Riboflavin-Responsive Exercise Intolerance. N. Engl. J. Med..

[182] Ghosh A., Trivedi P.P., Timbalia S.A., Griffin A.T., Rahn J.J., Chan S.S.L., Gohil V.M. (2014). Copper Supplementation Restores Cytochrome c Oxidase Assembly Defect in a Mitochondrial Disease Model of COA6 Deficiency. Hum. Mol. Genet..

[183] Ghosh A., Pratt A.T., Soma S., Theriault S.G., Griffin A.T., Trivedi P.P., Gohil V.M. (2016). Mitochondrial Disease Genes COA6, COX6B and SCO2 Have Overlapping Roles in COX2 Biogenesis. Hum. Mol. Genet..

[184] López-Martín J.M., Salviati L., Trevisson E., Montini G., DiMauro S., Quinzii C., Hirano M., Rodriguez-Hernandez A., Cordero M.D., Sánchez-Alcázar J.A. (2007). Missense Mutation of the COQ2 Gene Causes Defects of Bioenergetics and de Novo Pyrimidine Synthesis. Hum. Mol. Genet..

[185] (2013). Multiple-System Atrophy Research Collaboration Mutations in COQ2 in Familial and Sporadic Multiple-System Atrophy. N. Engl. J. Med..

[186] Desbats M.A., Morbidoni V., Silic-Benussi M., Doimo M., Ciminale V., Cassina M., Sacconi S., Hirano M., Basso G., Pierrel F. (2016). The COQ2 Genotype Predicts the Severity of Coenzyme Q10 Deficiency. Hum. Mol. Genet..

[187] Mollet J., Giurgea I., Schlemmer D., Dallner G., Chretien D., Delahodde A., Bacq D., de Lonlay P., Munnich A., Rötig A. (2007). Prenyldiphosphate Synthase, Subunit 1 (PDSS1) and OH-Benzoate Polyprenyltransferase (COQ2) Mutations in Ubiquinone Deficiency and Oxidative Phosphorylation Disorders. J. Clin. Invest..

[188] Salviati L., Trevisson E., Rodriguez Hernandez M.A., Casarin A., Pertegato V., Doimo M., Cassina M., Agosto C., Desbats M.A., Sartori G. (2012). Haploinsufficiency of COQ4 Causes Coenzyme Q10 Deficiency. J. Med. Genet..

[189] Brea-Calvo G., Haack T.B., Karall D., Ohtake A., Invernizzi F., Carrozzo R., Kremer L., Dusi S., Fauth C., Scholl-Bürgi S. (2015). COQ4 Mutations Cause a Broad Spectrum of Mitochondrial Disorders Associated with CoQ10 Deficiency. Am. J. Hum. Genet..

[190] Nguyen T.P.T., Casarin A., Desbats M.A., Doimo M., Trevisson E., Santos-Ocaña C., Navas P., Clarke C.F., Salviati L. (2014). Molecular Characterization of the Human COQ5 C-Methyltransferase in Coenzyme Q10 Biosynthesis. Biochim. Biophys. Acta.

[191] Heeringa S.F., Chernin G., Chaki M., Zhou W., Sloan A.J., Ji Z., Xie L.X., Salviati L., Hurd T.W., Vega-Warner V. (2011). COQ6 Mutations in Human Patients Produce Nephrotic Syndrome with Sensorineural Deafness. J. Clin. Invest..

[192] Doimo M., Trevisson E., Airik R., Bergdoll M., Santos-Ocaña C., Hildebrandt F., Navas P., Pierrel F., Salviati L. (2014). Effect of Vanillic Acid on COQ6 Mutants Identified in Patients with Coenzyme Q10 Deficiency. Biochim. Biophys. Acta.

[193] Xie L.X., Hsieh E.J., Watanabe S., Allan C.M., Chen J.Y., Tran U.C., Clarke C.F. (2011). Expression of the Human Atypical Kinase ADCK3 Rescues Coenzyme Q Biosynthesis and Phosphorylation of Coq Polypeptides in Yeast Coq8 Mutants. Biochim. Biophys. Acta.

[194] Vazquez Fonseca L., Doimo M., Calderan C., Desbats M.A., Acosta M.J., Cerqua C., Cassina M., Ashraf S., Hildebrandt F., Sartori G. (2018). Mutations in COQ8B (ADCK4) Found in Patients with Steroid-Resistant Nephrotic Syndrome Alter COQ8B Function. Hum. Mutat.

[195] He C.H., Black D.S., Allan C.M., Meunier B., Rahman S., Clarke C.F. (2017). Human COQ9 Rescues a Coq9 Yeast Mutant by Enhancing Coenzyme Q Biosynthesis from 4-Hydroxybenzoic Acid and Stabilizing the CoQ-Synthome. Front. Physiol..

[196] Duncan A.J., Bitner-Glindzicz M., Meunier B., Costello H., Hargreaves I.P., López L.C., Hirano M., Quinzii C.M., Sadowski M.I., Hardy J. (2009). A Nonsense Mutation in COQ9 Causes Autosomal-Recessive Neonatal-Onset Primary Coenzyme Q10 Deficiency: A Potentially Treatable Form of Mitochondrial Disease. Am. J. Hum. Genet..

[197] De Rocco D., Cerqua C., Goffrini P., Russo G., Pastore A., Meloni F., Nicchia E., Moraes C.T., Pecci A., Salviati L. (2014). Mutations of Cytochrome c Identified in Patients with Thrombocytopenia THC4 Affect Both Apoptosis and Cellular Bioenergetics. Biochim. Biophys. Acta.

[198] Uchiyama Y., Yanagisawa K., Kunishima S., Shiina M., Ogawa Y., Nakashima M., Hirato J., Imagawa E., Fujita A., Hamanaka K. (2018). A Novel CYCS Mutation in the α-Helix of the CYCS C-Terminal Domain Causes Non-Syndromic Thrombocytopenia. Clin. Genet..

[199] Wimplinger I., Morleo M., Rosenberger G., Iaconis D., Orth U., Meinecke P., Lerer I., Ballabio A., Gal A., Franco B. (2006). Mutations of the Mitochondrial Holocytochrome C-Type Synthase in X-Linked Dominant Microphthalmia with Linear Skin Defects Syndrome. Am. J. Hum. Genet..

[200] Wimplinger I., Shaw G.M., Kutsche K. (2007). HCCS Loss-of-Function Missense Mutation in a Female with Bilateral Microphthalmia and Sclerocornea: A Novel Gene for Severe Ocular Malformations?. Mol. Vis..

[201] Indrieri A., Conte I., Chesi G., Romano A., Quartararo J., Tatè R., Ghezzi D., Zeviani M., Goffrini P., Ferrero I. (2013). The Impairment of HCCS Leads to MLS Syndrome by Activating a Non-Canonical Cell Death Pathway in the Brain and Eyes. EMBO Mol. Med..

[202] Cruciat C.M., Hell K., Fölsch H., Neupert W., Stuart R.A. (1999). Bcs1p, an AAA-Family Member, Is a Chaperone for the Assembly of the Cytochrome Bc(1) Complex. EMBO J..

[203] Aghajanian S., Worrall D.M. (2002). Identification and Characterization of the Gene Encoding the Human Phosphopantetheine Adenylyltransferase and Dephospho-CoA Kinase Bifunctional Enzyme (CoA Synthase). Biochem. J..

[204] Di Meo I., Cavestro C., Pedretti S., Fu T., Ligorio S., Manocchio A., Lavermicocca L., Santambrogio P., Ripamonti M., Levi S. (2020). Neuronal Ablation of CoA Synthase Causes Motor Deficits, Iron Dyshomeostasis, and Mitochondrial Dysfunctions in a CoPAN Mouse Model. Int. J. Mol. Sci..

[205] Storici F., Resnick M.A. (2003). Delitto Perfetto Targeted Mutagenesis in Yeast with Oligonucleotides. Genet. Eng..

[206] Olsson A., Lind L., Thornell L.-E., Holmberg M. (2008). Myopathy with Lactic Acidosis Is Linked to Chromosome 12q23.3-24.11 and Caused by an Intron Mutation in the ISCU Gene Resulting in a Splicing Defect. Hum. Mol. Genet..

[207] Mochel F., Knight M.A., Tong W.-H., Hernandez D., Ayyad K., Taivassalo T., Andersen P.M., Singleton A., Rouault T.A., Fischbeck K.H. (2008). Splice Mutation in the Iron-Sulfur Cluster Scaffold Protein ISCU Causes Myopathy with Exercise Intolerance. Am. J. Hum. Genet..

[208] Kollberg G., Tulinius M., Melberg A., Darin N., Andersen O., Holmgren D., Oldfors A., Holme E. (2009). Clinical Manifestation and a New ISCU Mutation in Iron-Sulphur Cluster Deficiency Myopathy. Brain.

[209] Fukasawa Y., Tsuji J., Fu S.-C., Tomii K., Horton P., Imai K. (2015). MitoFates: Improved Prediction of Mitochondrial Targeting Sequences and Their Cleavage Sites. Mol. Cell. Proteom..

[210] Nasca A., Rizza T., Doimo M., Legati A., Ciolfi A., Diodato D., Calderan C., Carrara G., Lamantea E., Aiello C. (2017). Not Only Dominant, Not Only Optic Atrophy: Expanding the Clinical Spectrum Associated with OPA1 Mutations. Orphanet J. Rare Dis..

[211] Hatanaka T., Takemoto Y., Hashimoto M., Majima E., Shinohara Y., Terada H. (2001). Significant Expression of Functional Human Type 1 Mitochondrial ADP/ATP Carrier in Yeast Mitochondria. Biol. Pharm. Bull..

[212] Baruffini E., Ferrero I., Foury F. (2010). In Vivo Analysis of MtDNA Replication Defects in Yeast. Methods.

[213] Lodi T., Dallabona C., Nolli C., Goffrini P., Donnini C., Baruffini E. (2015). DNA Polymerase γ and Disease: What We Have Learned from Yeast. Front. Genet..

[214] Zakharov I.A., Yarovoy B.P. (1977). Cytoduction as a New Tool in Studying the Cytoplasmic Heredity in Yeast. Mol. Cell. Biochem..

[215] Foury F., Szczepanowska K. (2011). Antimutator Alleles of Yeast DNA Polymerase Gamma Modulate the Balance between DNA Synthesis and Excision. PLoS ONE.

[216] Barrientos A., Fontanesi F., Díaz F. (2009). Evaluation of the Mitochondrial Respiratory Chain and Oxidative Phosphorylation System Using Polarography and Spectrophotometric Enzyme Assays. Curr. Protoc Hum. Genet..

[217] Somlo M. (1968). Induction and Repression of Mitochondrial ATPase in Yeast. Eur. J. Biochem..

[218] Zorova L.D., Popkov V.A., Plotnikov E.Y., Silachev D.N., Pevzner I.B., Jankauskas S.S., Babenko V.A., Zorov S.D., Balakireva A.V., Juhaszova M. (2018). Mitochondrial Membrane Potential. Anal. Biochem..

[219] Zorov D.B., Juhaszova M., Sollott S.J. (2014). Mitochondrial Reactive Oxygen Species (ROS) and ROS-Induced ROS Release. Physiol. Rev..

[220] Müller M., Lu K., Reichert A.S. (2015). Mitophagy and Mitochondrial Dynamics in *Saccharomyces cerevisiae*. Biochim. Biophys. Acta.

[221] Dujon B., Slonimski P.P., Weill L. (1974). Mitochondrial Genetics IX: A Model for Recombination and Segregation of Mitochondrial Genomes in *Saccharomyces cerevisiae*. Genetics.

[222] Hori A., Yoshida M., Shibata T., Ling F. (2009). Reactive Oxygen Species Regulate DNA Copy Number in Isolated Yeast Mitochondria by Triggering Recombination-Mediated Replication. Nucleic Acids Res..

[223] Chen X.J., Butow R.A. (2005). The Organization and Inheritance of the Mitochondrial Genome. Nat. Rev. Genet..

[224] Lipinski K.A., Kaniak-Golik A., Golik P. (2010). Maintenance and Expression of the S. Cerevisiae Mitochondrial Genome--from Genetics to Evolution and Systems Biology. Biochim. Biophys. Acta.

[225] Kucej M., Butow R.A. (2007). Evolutionary Tinkering with Mitochondrial Nucleoids. Trends Cell Biol..

[226] Diffley J.F., Stillman B. (1991). A Close Relative of the Nuclear, Chromosomal High-Mobility Group Protein HMG1 in Yeast Mitochondria. Proc. Natl. Acad. Sci. USA.

[227] Solieri L. (2010). Mitochondrial Inheritance in Budding Yeasts: Towards an Integrated Understanding. Trends Microbiol..

[228] Westermann B. (2014). Mitochondrial Inheritance in Yeast. Biochim. Biophys. Acta.

[229] Miyakawa I. (2017). Organization and Dynamics of Yeast Mitochondrial Nucleoids. Proc. Jpn. Acad. Ser. B Phys. Biol. Sci..

[230] Sherman F. (1963). Respiration-Deficient Mutants of Yeast. I. Genetics. Genetics.

[231] Dujon B. (2020). Mitochondrial Genetics Revisited. Yeast.

[232] Contamine V., Picard M. (2000). Maintenance and Integrity of the Mitochondrial Genome: A Plethora of Nuclear Genes in the Budding Yeast. Microbiol. Mol. Biol. Rev..

[233] Dujon B. (1981). Mitochondrial genetics and functions. The Molecular Biology of the Yeast Saccharomyces. Life Cycle and Inheritance.

[234] Birky C.W. (2001). The Inheritance of Genes in Mitochondria and Chloroplasts: Laws, Mechanisms, and Models. Annu. Rev. Genet..

[235] Shibata T., Ling F. (2007). DNA Recombination Protein-Dependent Mechanism of Homoplasmy and Its Proposed Functions. Mitochondrion.

[236] Gonzalez-Hunt C.P., Rooney J.P., Ryde I.T., Anbalagan C., Joglekar R., Meyer J.N. (2016). PCR-Based Analysis of Mitochondrial DNA Copy Number, Mitochondrial DNA Damage, and Nuclear DNA Damage. Curr. Protoc. Toxicol..

[237] Baruffini E., Ferrari J., Dallabona C., Donnini C., Lodi T. (2015). Polymorphisms in DNA Polymerase γ Affect the MtDNA Stability and the NRTI-Induced Mitochondrial Toxicity in *Saccharomyces cerevisiae*. Mitochondrion.

[238] Chan D.C. (2006). Mitochondrial fusion and fission in mammals. Annu Rev. Cell Dev. Biol..

[239] Westermann B. (2010). Mitochondrial fusion and fission in cell life and death. Nat. Rev. Mol. Cell Biol..

[240] Rapaport D., Brunner M., Neupert W., Westermann B. (1998). Fzo1p is a mitochondrial outer membrane protein essential for the biogenesis of functional mitochondria in *Saccharomyces cerevisiae*. J. Biol. Chem..

[241] Zick M., Duvezin-Caubet S., Schäfer A., Vogel F., Neupert W., Reichert A.S. (2009). Distinct roles of the two isoforms of the dynamin-like GTPase Mgm1 in mitochondrial fusion. FEBS Lett..

[242] Chen H., Vermulst M., Wang Y.E., Chomyn A., Prolla T.A., McCaffery J.M., Chan D.C. (2010). Mitochondrial fusion is required for mtDNA stability in skeletal muscle and tolerance of mtDNA mutations. Cell.

[243] Kraus F., Roy K., Pucadyil T.J., Ryan M.T. (2021). Function and regulation of the divisome for mitochondrial fission. Nature.

[244] Skowronek E., Grzechnik P., Späth B., Marchfelder A., Kufel J. (2014). TRNA 3’ Processing in Yeast Involves TRNase Z, Rex1, and Rrp6. RNA.

[245] Montanari A., Besagni C., De Luca C., Morea V., Oliva R., Tramontano A., Bolotin-Fukuhara M., Frontali L., Francisci S. (2008). Yeast as a Model of Human Mitochondrial TRNA Base Substitutions: Investigation of the Molecular Basis of Respiratory Defects. RNA.

[246] Funes S., Herrmann J.M. (2007). Analysis of Mitochondrial Protein Synthesis in Yeast. Methods Mol. Biol..

[247] Marres C.A., de Vries S., Grivell L.A. (1991). Isolation and Inactivation of the Nuclear Gene Encoding the Rotenone-Insensitive Internal NADH: Ubiquinone Oxidoreductase of Mitochondria from *Saccharomyces cerevisiae*. Eur. J. Biochem..

[248] Schägger H., von Jagow G. (1991). Blue Native Electrophoresis for Isolation of Membrane Protein Complexes in Enzymatically Active Form. Anal. Biochem..

[249] Nijtmans L.G.J., Henderson N.S., Holt I.J. (2002). Blue Native Electrophoresis to Study Mitochondrial and Other Protein Complexes. Methods.

[250] Jha P., Wang X., Auwerx J. (2016). Analysis of Mitochondrial Respiratory Chain Supercomplexes Using Blue Native Polyacrylamide Gel Electrophoresis (BN-PAGE). Curr. Protoc. Mouse Biol..

[251] Dallabona C., Marsano R.M., Arzuffi P., Ghezzi D., Mancini P., Zeviani M., Ferrero I., Donnini C. (2010). Sym1, the Yeast Ortholog of the MPV17 Human Disease Protein, Is a Stress-Induced Bioenergetic and Morphogenetic Mitochondrial Modulator. Hum. Mol. Genet..

[252] Pir P., Gutteridge A., Wu J., Rash B., Kell D.B., Zhang N., Oliver S.G. (2012). The Genetic Control of Growth Rate: A Systems Biology Study in Yeast. BMC Syst. Biol..

[253] Deutschbauer A.M., Jaramillo D.F., Proctor M., Kumm J., Hillenmeyer M.E., Davis R.W., Nislow C., Giaever G. (2005). Mechanisms of Haploinsufficiency Revealed by Genome-Wide Profiling in Yeast. Genetics.

[254] Le Roux B., Lenaers G., Zanlonghi X., Amati-Bonneau P., Chabrun F., Foulonneau T., Caignard A., Leruez S., Gohier P., Procaccio V. (2019). OPA1: 516 Unique Variants and 831 Patients Registered in an Updated Centralized Variome Database. Orphanet J. Rare Dis..

[255] Rahman S., Copeland W.C. (2019). POLG-Related Disorders and Their Neurological Manifestations. Nat. Rev. Neurol..

[256] Viscomi C., Zeviani M. (2020). Strategies for Fighting Mitochondrial Diseases. J. Intern. Med..

[257] Rötig A., de Lonlay P., Chretien D., Foury F., Koenig M., Sidi D., Munnich A., Rustin P. (1997). Aconitase and Mitochondrial Iron-Sulphur Protein Deficiency in Friedreich Ataxia. Nat. Genet..

[258] Cotticelli M.G., Rasmussen L., Kushner N.L., McKellip S., Sosa M.I., Manouvakhova A., Feng S., White E.L., Maddry J.A., Heemskerk J. (2012). Primary and Secondary Drug Screening Assays for Friedreich Ataxia. J. Biomol. Screen.

[259] Couplan E., Aiyar R.S., Kucharczyk R., Kabala A., Ezkurdia N., Gagneur J., St Onge R.P., Salin B., Soubigou F., Le Cann M. (2011). A Yeast-Based Assay Identifies Drugs Active against Human Mitochondrial Disorders. Proc. Natl. Acad. Sci. USA.

[260] Hoon S., St Onge R.P., Giaever G., Nislow C. (2008). Yeast Chemical Genomics and Drug Discovery: An Update. Trends Pharmacol. Sci..

[261] Aiyar R.S., Bohnert M., Duvezin-Caubet S., Voisset C., Gagneur J., Fritsch E.S., Couplan E., von der Malsburg K., Funaya C., Soubigou F. (2014). Mitochondrial Protein Sorting as a Therapeutic Target for ATP Synthase Disorders. Nat. Commun..

[262] Panozzo C., Laleve A., Tribouillard-Tanvier D., Ostojić J., Sellem C.H., Friocourt G., Bourand-Plantefol A., Burg A., Delahodde A., Blondel M. (2017). Chemicals or Mutations That Target Mitochondrial Translation Can Rescue the Respiratory Deficiency of Yeast Bcs1 Mutants. Biochim. Biophys. Acta Mol. Cell Res..

[263] Delettre C., Lenaers G., Griffoin J.M., Gigarel N., Lorenzo C., Belenguer P., Pelloquin L., Grosgeorge J., Turc-Carel C., Perret E. (2000). Nuclear Gene OPA1, Encoding a Mitochondrial Dynamin-Related Protein, Is Mutated in Dominant Optic Atrophy. Nat. Genet..

[264] Alexander C., Votruba M., Pesch U.E., Thiselton D.L., Mayer S., Moore A., Rodriguez M., Kellner U., Leo-Kottler B., Auburger G. (2000). OPA1, Encoding a Dynamin-Related GTPase, Is Mutated in Autosomal Dominant Optic Atrophy Linked to Chromosome 3q28. Nat. Genet..

[265] Amati-Bonneau P., Milea D., Bonneau D., Chevrollier A., Ferré M., Guillet V., Gueguen N., Loiseau D., de Crescenzo M.-A.P., Verny C. (2009). OPA1-Associated Disorders: Phenotypes and Pathophysiology. Int. J. Biochem. Cell Biol..

[266] Jones B.A., Fangman W.L. (1992). Mitochondrial DNA Maintenance in Yeast Requires a Protein Containing a Region Related to the GTP-Binding Domain of Dynamin. Genes Dev..

[267] Aleo S.J., Del Dotto V., Fogazza M., Maresca A., Lodi T., Goffrini P., Ghelli A., Rugolo M., Carelli V., Baruffini E. (2021). Drug Repositioning as a Therapeutic Strategy for Neurodegenerations Associated with OPA1 Mutations. Hum. Mol. Genet..

[268] Pitayu L., Baruffini E., Rodier C., Rötig A., Lodi T., Delahodde A. (2016). Combined Use of *Saccharomyces cerevisiae*, Caenorhabditis Elegans and Patient Fibroblasts Leads to the Identification of Clofilium Tosylate as a Potential Therapeutic Chemical against POLG-Related Diseases. Hum. Mol. Genet..

[269] Facchinello N., Laquatra C., Locatello L., Beffagna G., Brañas Casas R., Fornetto C., Dinarello A., Martorano L., Vettori A., Risato G. (2021). Efficient Clofilium Tosylate-Mediated Rescue of POLG-Related Disease Phenotypes in Zebrafish. Cell Death Dis..

[270] Tigano M., Ruotolo R., Dallabona C., Fontanesi F., Barrientos A., Donnini C., Ottonello S. (2015). Elongator-Dependent Modification of Cytoplasmic TRNALysUUU Is Required for Mitochondrial Function under Stress Conditions. Nucleic Acids Res..

[271] Varghese F., Atcheson E., Bridges H.R., Hirst J. (2015). Characterization of clinically identified mutations in NDUFV1, the flavin-binding subunit of respiratory complex I, using a yeast model system. Hum. Mol. Genet..

